# Fission yeast Rad8/HLTF facilitates Rad52-dependent chromosomal rearrangements through PCNA lysine 107 ubiquitination

**DOI:** 10.1371/journal.pgen.1009671

**Published:** 2021-07-22

**Authors:** Jie Su, Ran Xu, Piyusha Mongia, Naoko Toyofuku, Takuro Nakagawa

**Affiliations:** Department of Biological Sciences, Graduate School of Science, Osaka University, Toyonaka, Japan; University of Illinois College of Pharmacy, UNITED STATES

## Abstract

Gross chromosomal rearrangements (GCRs), including translocation, deletion, and inversion, can cause cell death and genetic diseases such as cancer in multicellular organisms. Rad51, a DNA strand exchange protein, suppresses GCRs by repairing spontaneous DNA damage through a conservative way of homologous recombination, gene conversion. On the other hand, Rad52 that catalyzes single-strand annealing (SSA) causes GCRs using homologous sequences. However, the detailed mechanism of Rad52-dependent GCRs remains unclear. Here, we provide genetic evidence that fission yeast Rad8/HLTF facilitates Rad52-dependent GCRs through the ubiquitination of lysine 107 (K107) of PCNA, a DNA sliding clamp. In *rad51Δ* cells, loss of Rad8 eliminated 75% of the isochromosomes resulting from centromere inverted repeat recombination, showing that Rad8 is essential for the formation of the majority of isochromosomes in *rad51Δ* cells. Rad8 HIRAN and RING finger mutations reduced GCRs, suggesting that Rad8 facilitates GCRs through 3’ DNA-end binding and ubiquitin ligase activity. Mms2 and Ubc4 but not Ubc13 ubiquitin-conjugating enzymes were required for GCRs. Consistent with this, mutating PCNA K107 rather than the well-studied PCNA K164 reduced GCRs. Rad8-dependent PCNA K107 ubiquitination facilitates Rad52-dependent GCRs, as PCNA K107R, *rad8*, and *rad52* mutations epistatically reduced GCRs. In contrast to GCRs, PCNA K107R did not significantly change gene conversion rates, suggesting a specific role of PCNA K107 ubiquitination in GCRs. PCNA K107R enhanced temperature-sensitive growth defects of DNA ligase I *cdc17-K42* mutant, implying that PCNA K107 ubiquitination occurs when Okazaki fragment maturation fails. Remarkably, K107 is located at the interface between PCNA subunits, and an interface mutation D150E bypassed the requirement of PCNA K107 and Rad8 ubiquitin ligase for GCRs. These data suggest that Rad8-dependent PCNA K107 ubiquitination facilitates Rad52-dependent GCRs by changing the PCNA clamp structure.

## Author summary

Gross chromosomal rearrangements (GCRs), including translocation, can alter gene dosage and activity, resulting in genetic diseases such as cancer. However, GCRs can occur by some enzymes, including Rad52 recombinase, and result in chromosomal evolution. Therefore, GCRs are not only pathological but also physiological phenomena from an evolutionary point of view. However, the detailed mechanism of GCRs remains unclear. Here, using fission yeast, we show that the homolog of human HLTF, Rad8 causes GCRs through noncanonical ubiquitination of proliferating cellular nuclear antigen (PCNA) at a lysine 107 (K107). Rad51, a DNA strand exchange protein, suppresses the formation of isochromosomes whose arms mirror each another and chromosomal truncation. We found that, like Rad52, Rad8 is required for isochromosome formation but not chromosomal truncation in *rad51Δ* cells, showing a specific role of Rad8 in homology-mediated GCRs. Mutations in Rad8 ubiquitin E3 ligase RING finger domain, Mms2-Ubc4 ubiquitin-conjugating enzymes, and PCNA K107 reduced GCRs in *rad51Δ* cells, suggesting that Rad8-Mms2-Ubc4-dependent PCNA K107 ubiquitination facilitates GCRs. PCNA trimers form a DNA sliding clamp. The K107 residue is located at the PCNA-PCNA interface, and an interface mutation D150E restored GCRs in PCNA K107R mutant cells. This study provides genetic evidence that Rad8-dependent PCNA K107 ubiquitination facilitates GCRs by changing the PCNA clamp structure.

## Introduction

Faithful repair of DNA damage, such as DNA double-strand breaks (DSBs), is critical to maintaining genome integrity [1,2]. Homologous recombination is considered an error-free DSB repair mechanism, as it uses intact DNA as the template. However, when accompanied by crossover or break-induced replication, nonallelic homologous recombination between repetitive sequences in a genome results in gross chromosomal rearrangements (GCRs), including translocation, deletion, and inversion [3,4]. Isochromosomes, whose arms mirror each other, are the GCR products formed by recombination between inverted repeats around centromeres [5]. Replication fork stalling can cause GCR events without DSB formation [6]. Replication forks reassembled by homologous recombination following fork stalling are prone to switch template strands and form acentric and dicentric isochromosomes using nearby inverted repeats [7]. GCRs can cause cell death and genetic diseases, including cancer [8]. On the other hand, GCRs can also foster evolution of living organisms. However, the detailed mechanism of GCRs remains unclear.

Rad51 and Rad52 are evolutionarily conserved recombination enzymes that have pivotal roles in distinct recombination pathways [9,10]. Rad51 preferentially promotes conservative recombination, gene conversion [11–13]. Rad51 binds single-stranded DNA (ssDNA) and catalyzes DNA strand exchange with homologous double-stranded DNA (dsDNA) [14]. Mammalian BRCA1 and BRCA2 facilitate Rad51-dependent recombination. BRCA gene mutations increase GCRs and predispose the carriers to hereditary breast and ovarian cancer [15], demonstrating that Rad51-dependent recombination safeguards genome integrity and prevents tumorigenesis. Although yeast Rad52 mediates Rad51 loading onto RPA-coated ssDNA, human Rad52 has no such activity, and BRCA2 mediates Rad51 loading [14]. In contrast to the mediator function, both yeast and human Rad52 proteins catalyze single-strand annealing (SSA) by which complementary ssDNA strands are annealed to form dsDNA [16–18]. For the sake of simplicity, Rad52-dependent SSA that occurs independently of Rad51 is designated Rad52-dependent recombination in this paper. Rad52 knockout mice show only a mild defect in DNA recombinational repair and are not predisposed to cancer [19]. However, Rad52 deficiency is synthetic lethal with BRCA mutations [20,21]. Rad52 N-terminal domain that retains SSA activity is sufficient to restore cell growth of the double mutants [22], showing the importance of Rad52-dependent recombination in cells defective in Rad51-dependent recombination. Consistent with this idea, yeast *rad52Δ* cells exhibit more severe DNA repair and recombination defects than *rad51Δ* cells [3]. Previously, we have shown in fission yeast that loss of Rad51 reduces gene conversion between centromere inverted repeats and increases isochromosome formation in a manner dependent on Mus81 crossover-specific endonuclease [11,23]. Loss of Rad52 partially reduces isochromosome formation in *rad51Δ* cells [24], showing that isochromosomes are produced in both Rad52-dependent and Rad52-independent manners. In budding yeast, mutating the evolutionarily conserved arginine 70 (R70) in the N-terminal DNA-binding domain of Rad52 impairs *in vitro* SSA activity but not the mediator function [25]. Consistent with this, the *rad52-R70K* mutant phenotype in DNA recombination and repair is less severe than a *rad52* null mutant [26]. Fission yeast Rad52 R45 is the counterpart of budding yeast Rad52 R70. The fission yeast *rad52-R45K* mutation also impairs *in vitro* SSA activity and causes less severe phenotypes than *rad52* deletion [24]. The *rad52-R45K* mutation reduces isochromosome formation in *rad51Δ* cells to the same extent as *rad52Δ* [24], suggesting that the SSA activity of Rad52 is responsible for isochromosome formation. At fission yeast centromeres, Rad51-dependent recombination predominates, and Rad52-dependent recombination is suppressed [27]. Intriguingly, a mutation in DNA polymerase α (Pol α), required for lagging-strand synthesis, increases Rad52-dependent recombination without changing the total rate of recombination at centromeres [24], suggesting that the formation of ssDNA gaps during DNA replication might be a rate-limiting step of Rad52-dependent recombination. Rad52-dependent recombination leads to not only GCRs but also gene conversion in the absence of Rad51. Msh2-Msh3 MutS-homologs [28] and Mus81 crossover-specific endonuclease [29,30] are required for the GCR but not gene conversion branch of Rad52-dependent recombination that occurs in the absence of Rad51 [11,24], suggesting that Msh2-Msh3 and Mus81 resolve joint molecules formed by Rad52 specifically into GCR products. However, how Rad52-dependent recombination differentiates into GCRs is still unclear.

Proliferating cell nuclear antigen (PCNA) protein interacts with each other and forms a homotrimeric DNA clamp that serves as a landing pad for the factors related to replication and repair [31,32]. Replication factor C (RFC) complexes load the PCNA clamp onto DNA at primer ends [33]. After ligation of Okazaki fragments, RFC-like complexes containing Elg1 unload PCNA from DNA [34,35]. Post-translational modifications of PCNA are critical in regulating its function [36]. Among them, the ubiquitination of PCNA has been most well studied [37]. Rad18 ubiquitin ligase (E3) and Rad6 ubiquitin-conjugating enzyme (E2) catalyze PCNA K164 mono-ubiquitination to recruit translesion synthesis polymerases, including DNA Pol η. Depending on the mono-ubiquitination, budding yeast Rad5 (E3) and Mms2-Ubc13 (E2) catalyze K164 poly-ubiquitination to promote template switching, a recombination-mediated damage bypass pathway [38]. Fission yeast Rad8 and human HLTF are homologs of budding yeast Rad5. They are unique among ubiquitin ligases as they contain HIRAN and Snf2/Swi2 translocase domains besides the RING finger E2 binding domain [37,39,40]. HIRAN is a modified oligonucleotide/oligosaccharide (OB) fold domain that binds 3’ DNA-ends [41–43]. Interestingly, in the *cdc9* mutant strain of DNA ligase I, essential for Okazaki fragment ligation, budding yeast Rad5 (E3) ubiquitinates PCNA at K107 rather than K164 [44,45]. K107 ubiquitination requires Mms2-Ubc4 (E2) but not Mms2-Ubc13 (E2) and occurs independently of Rad18 (E3) or Rad6 (E2). K107 ubiquitination is required for the full activation of a checkpoint kinase in *cdc9* mutants. However, it is unknown how K107 ubiquitination affects PCNA structure and function.

Here, we provide genetic evidence that fission yeast Rad8 (E3) and Mms2-Ubc4 (E2) ubiquitinate PCNA at K107 to facilitate Rad52-dependent GCRs. Loss of Rad8 reduced isochromosome formation but not chromosomal truncation in *rad51Δ* cells. Mutation in Rad8 HIRAN or RING finger but not Snf2/Swi2 translocase domain reduced GCRs. *mms2* and *ubc4* but not *ubc13*; PCNA K107R but not K164R reduced GCRs in *rad51Δ* cells, suggesting that the ubiquitination of K107 but not a canonical site K164 is required for GCRs. The epistatic analysis showed that Rad8 and PCNA K107 play a crucial role in the GCR branch of Rad52-dependent recombination. K107 is located at the interface between PCNA subunits, suggesting that K107 ubiquitination weakens the PCNA-PCNA interaction to cause GCRs. Remarkably, an interface mutation D150E [46,47] indeed bypassed the requirement of Rad8 ubiquitin ligase and PCNA K107 for GCRs. These data suggest that PCNA K107 ubiquitination changes the PCNA clamp structure to facilitate Rad52-dependent GCRs.

## Results

### Fission yeast Rad8 facilitates isochromosome formation but not chromosomal truncation

**Fig 1:**
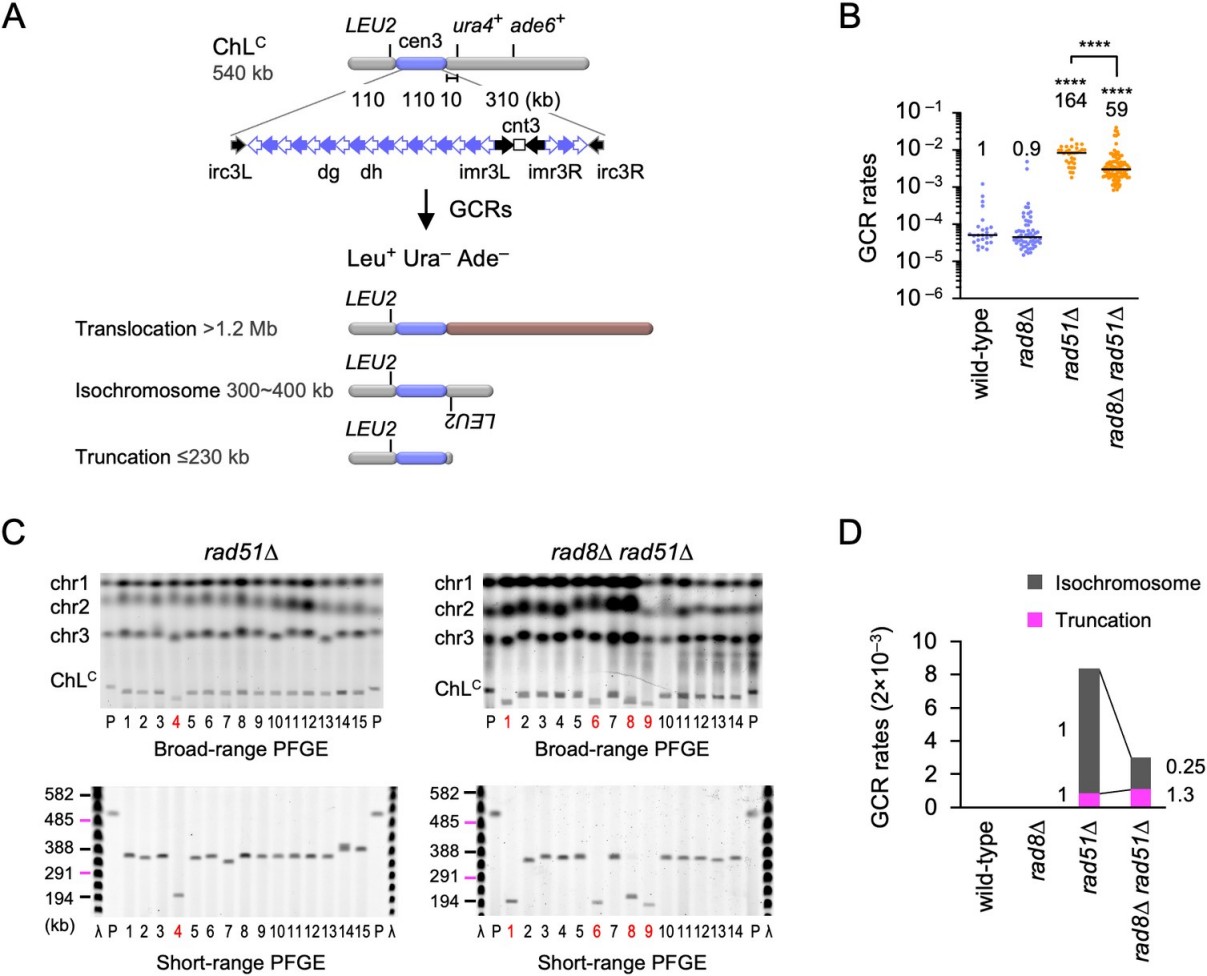
Fission yeast Rad8 facilitates isochromosome formation. (A) The ChL^C^ chromosome contains an entire region of centromere 3 (cen3) and three genetic markers: *LEU2*, *ura4*^+^, and *ade6*^+^. Gross chromosomal rearrangements (GCRs) associated with loss of both *ura4*^+^ and *ade6*^+^ result in the formation of Leu^+^ Ura^−^ Ade^−^ cells. The structure and the length of three GCR types: translocation, isochromosome, and truncation are shown. (B) GCR rates of the wild-type, *rad8Δ*, *rad51Δ*, and *rad8Δ rad51Δ* strains (TNF5369, 5549, 5411, and 5644, respectively). Each dot represents an independent experimental value obtained from an independent colony. Black bars indicate medians. Rates relative to that of wild type are shown on the top of each cluster of dots. Statistical analyses between the wild-type and mutant strains, and that between *rad51Δ* and *rad8Δ rad51Δ* strains were performed by the two-tailed Mann-Whitney test. **** *P* < 0.0001. (C) Chromosomal DNAs prepared from the parental (P) and independent GCR clones of *rad51Δ* and *rad8Δ rad51Δ* strains were separated by broad- and short-range PFGE (top and bottom rows, respectively). Positions of chr1, chr2, chr3, and ChL^C^ (5.7, 4.6, ~3.5, and 0.5 Mb, respectively) are indicated on the left of broad-range PFGE panels. Sizes of Lambda (λ) ladders (ProMega-Markers, Madison, Wisconsin, G3011) are displayed on the left of short-range PFGE panels. Sample number of truncations are highlighted in red. (D) Rates of each GCR type. Rates relative to those of *rad51Δ* are indicated. Numerical source data underlying the graphs shown in (B) and (D) are provided respectively in Tables A and B in S1 File.

Loss of Rad8 increases DNA damage sensitivity of *rad51Δ* cells [39,48], raising the possibility that Rad8 is involved in GCRs that occur independently of Rad51. To test this, we disrupted the *rad8* gene in the fission yeast cell harbouring the extra-chromosome ChL^C^ derived from chromosome 3 (chr3) (Fig 1A). ChL^C^ retains an entire region of centromere 3 (cen3) that consists of a unique sequence, cnt3, surrounded by inverted repeats: imr3, dg, dh, and irc3, and contains three genetic markers: *LEU2*, *ura4*^+^, and *ade6*^+^ [11,23]. As ChL^C^ is dispensable for cell growth, one can detect otherwise lethal GCRs using ChL^C^. To detect Leu^+^ Ura^−^ Ade^−^ GCR clones that have lost both *ura4*^+^ and *ade6*^+^, cells were grown in Edinburgh minimum media supplemented with uracil and adenine (EMM+UA) and plated on FOA+UA media containing 5-fluoroorotic acid (5-FOA) which is toxic to *ura4*^+^ cells. Leu^+^ Ura^−^ colonies formed on FOA+UA plates were transferred to EMM+U to inspect adenine auxotrophy. Fluctuation analysis showed that *rad8Δ* did not significantly change spontaneous GCR rates in wild-type cells (Fig 1B). However, *rad8Δ* partially but significantly reduced GCRs in *rad51Δ* cells (Fig 1B), indicating that Rad8 facilitates GCRs that occur independently of Rad51.

Three types of GCRs have been detected using the extra-chromosome: translocation, isochromosome, and truncation, which can be distinguished by their lengths [11,23,24,49–51] (Fig 1A). Among them, isochromosomes are formed by recombination between inverted repeats at centromeres. To determine GCR types Rad8 causes, chromosomal DNAs were prepared from the parental and independent GCR clones, resolved by pulsed-field gel electrophoresis (PFGE) under two different conditions (broad- and short-range PFGE), and stained with ethidium bromide (EtBr) (Figs 1C and S1; results for wild-type and *rad8Δ* and additional results for *rad51Δ* and *rad8 rad51Δ*). As previously observed [24], isochromosomes and a small number of translocations were detected in wild-type cells (Table 1). *rad8Δ* did not significantly change the GCR types in wild-type cells (*P* = 0.6, the two-tailed Fisher’s exact test). As previously observed [23,24,50], *rad51Δ* cells produced isochromosomes and a small number of truncations (Fig 1C, sample #4). Given elevated GCR rates (Fig 1B), these results show that *rad51Δ* increases isochromosome formation and chromosomal truncation. In *rad51Δ* cells, *rad8Δ* increased the proportion of truncations from 10 to 37% (Table 1; *P* = 0.030, the two-tailed Fisher’s exact test). To obtain the rate of each GCR type (Fig 1D), we multiplied the total GCR rate (Fig 1B) by the proportion of each GCR type (Table 1). Loss of Rad8 eliminated 75% of the isochromosomes produced in *rad51Δ* cells. On the other hand, Rad8 was dispensable for chromosomal truncation. These data demonstrate that Rad8 is specifically involved in the major pathway of isochromosome formation in *rad51Δ* cells.

**Table 1:** GCR types.

	translocation	isochromosome	truncation*	total
wild type	1 (3%)	31 (97%)	0	32
*rad8Δ*	2 (7%)	28 (93%)	0	30
*rad51Δ*	0	27 (90%)	3 [2] (10%)	30
*rad8Δ rad51Δ*	0	19 (63%)	11 [9] (37%)	30

Percentages of each GCR type are shown in ().

*The numbers of the truncation products whose breakpoints are present in centromere repeats are shown in [].

It has been shown that truncation ends are healed by *de novo* telomere addition [52–54]. To determine the chromosomal sites to which telomere sequences have been added, we recovered chromosomal truncations from agarose gels and amplified the breakpoints using a pair of ChL^C^ and telomere primers: M13R-C19 or M13R-T1 [55]. DNA sequencing of the PCR products revealed that telomere sequences (G_2-5_TTAC (A) (C)) [56] were added either within or outside centromere repeats (Tables 1 and 2). Only 0~3-bp overlaps were detected between ChL^C^ and telomere sequences around the breakpoints, suggesting that no extensive annealing with telomere RNA is required to initiate telomere addition. No apparent differences were detected between *rad51Δ* and *rad8Δ rad51Δ*. Together, these results show that Rad8 is required for homology-mediated GCRs resulting in isochromosome formation but dispensable for *de novo* telomere addition resulting in chromosomal truncation.

**Table 2:** Breakpoints of chromosomal truncations.

strain	clone	ChL^C^ primer	region	genome position (bp)		from 5’ to 3’
*rad51Δ*	4	4B3-1	arm	1,145,112	truncation:	ACTCCCTTCCCATT|**G**|**GTTACAGGGGTTACGGTTACACGG**
					original:	ACTCCCTTCCCATT|G|ATAAAGTCATCGGTTGATAAACAT
	20	otr3-1	dg	1,113,886	truncation:	GAGATTGAGTAAGA|**A**|**CGGTTACAGGTTACAGGTTACGGT**
					original:	GAGATTGAGTAAGA|A|GTGTTATGGAATAAGCAAAGTTAA
	23	otr3-tel3	irc3R	1,138,601	truncation:	AAGTGCACGAGGGT|**TT**|**ACGGTTACAGGTTACCGGTTACA**
					original:	AAGTGCACGAGGGT|TT|TGAGATGCAACGTTATTCGCTGT
*rad8Δ rad51Δ*	1	otr3-tel	irc3R	1,137,505	truncation:	AAAACCGATATGTG|**CGGTTACGGTTACAGGGGGTTACAG**
					original:	AAAACCGATATGTG|GGTTGCAAAAGATAAGCAGTCACCG
	6	otr3-1	dh	1,109,718	truncation:	GGGTTATCTCATAT|**CGG**|**TTACACGGTTACAGGTTACGGT**
					original:	GGGTTATCTCATAT|CGG|GAAACACTTTCTGCCACTTTTA
	8	set9-F1	arm	1,154,318	truncation:	TTTGTGTCGCAGAG|**TACGGTTACAGGTTACAGGTTACAG**
					original:	TTTGTGTCGCAGAG|ACATACTATGCGAGCTGGTAACTAA
	9	cnt3-r1	imr3R	1,104,238	truncation:	TGGCTGCTTCCTCT|**TTA**|**CGGTTACAGGTTACAGGGGGTT**
					original:	TGGCTGCTTCCTCT|TTA|ATTTAAATAAATAGTTTAGCAA
	15	otr3-1	dg	1,112,953	truncation:	TTTTTCCTCTTCGT|**TTACGGTTACAGGTTACAGGGTTAC**
					original:	TTTTTCCTCTTCGT|ATGGTGTGTAAACTGAATGGAAACG
	18	imr3-tel1	imr3R	1,104,723	truncation:	TTGTTGACAAATGG|**C**|**GGTTACAGGTTACAGGGTTACGGT**
					original:	TTGTTGACAAATGG|C|AAATACTCAAGCCAATAAAGAAAT
	20	4B3-1	arm	1,145,527	truncation:	TTTCACACTTCTGG|**TAC**|**GGTTACAGGGGTTACACGGTTA**
					original:	TTTCACACTTCTGG|TAC|CAAAATTCAAAAGCACCTAGCG
	21	otr3-1	irc3R	1,137,822	truncation:	GTGGTGGTTATGGA|**G**|**GTACGGTTACAGGGTTACGGTTAC**
					original:	GTGGTGGTTATGGA|G|TTTAACAAACAAGAAAAATGAACA
	23	GCR 26F	irc3R	1,140,586	truncation:	AAATAATCCAAATT|**CG**|**GTTACAGGTTACAGGGGTTACAG**
					original:	AAATAATCCAAATT|CG|ACTACTCATTAAGTATGCAGCAA
	27	otr3-tel3	dh	1,115,800	truncation:	CCCGCCCAGTGGAT|**G**|**GGGGTTACACGGTTACGGTTACAG**
					original:	CCCGCCCAGTGGAT|G|CTTCTGTGAATACACAAAAGTTTT
	29	cnt3-r1	dh	1,110,116	truncation:	TATCGTTTGTGTTT|**GGTTACAGGTTACGGTTACAGGTTA**
					original:	TATCGTTTGTGTTT|ATAAATCATCAGCCTCTCTCTATAT

Telomere repeat sequences are bold. The nucleotides overlapping between ChL^C^ and telomere sequences are placed between lines. The breakpoint positions are expressed as genome positions of chromosome 3.

### Rad8 HIRAN and RING finger domains are required for GCRs

**Fig 2:**
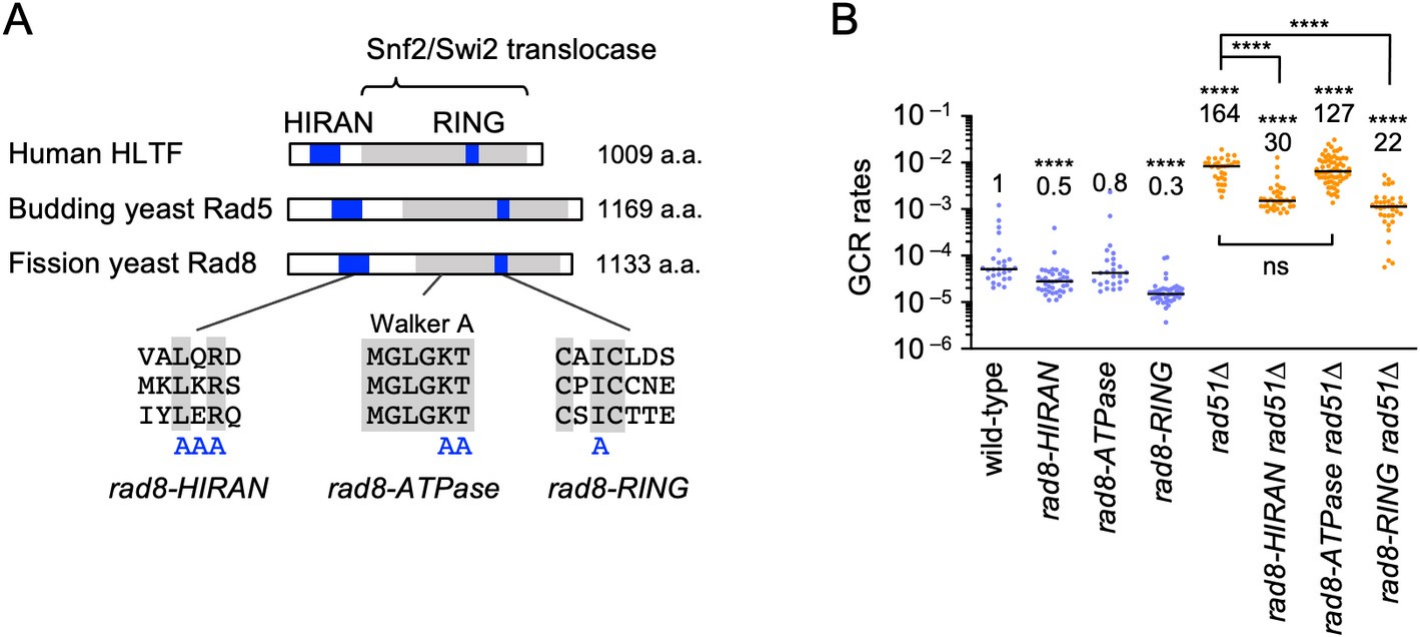
Rad8 HIRAN and RING domains are required for GCRs. (A) A schematic diagram showing the HIRAN, Snf2/Swi2 translocase, and RING finger domains of human HLTF, budding yeast Rad5, and fission yeast Rad8. Amino acid residues substituted for alanine in the *rad8-HIRAN*, *rad8-ATPase*, and *rad8-RING* mutations are indicated. (B) GCR rates of the wild-type, *rad8-HIRAN*, *rad8-ATPase*, *rad8-RING*, *rad51Δ*, *rad8-HIRAN rad51Δ*, *rad8-ATPase rad51Δ*, and *rad8-RING rad51Δ* strains (TNF5369, 6205, 6203, 6207, 5411, 6217, 6231, and 6219, respectively). The two-tailed Mann-Whitney test. Non-significant (ns) *P* > 0.05; **** *P* < 0.0001. Numerical source data underlying the graph shown in (B) are provided in Table C in S1 File.

Rad8 and its homologs contain HIRAN, Snf2/Swi2 translocase, and RING finger domains (Fig 2A, top). To gain insight into how Rad8 facilitates GCRs, we introduced alanine (A) substitution into each domain (Fig 2A, bottom). The *rad8-HIRAN* mutation alters the residues forming the 3’ DNA-end binding pocket [41–43]. The *rad8-ATPase* mutation alters the conserved residues in the ATP-binding Walker A motif [57]. The *rad8-RING* mutation changes a residue involved in the interaction with ubiquitin-conjugating enzymes [58,59]. *rad8-ATPase* did not significantly change GCR rates in both wild-type and *rad51Δ* cells (Fig 2B), indicating that Rad8 translocase activity is not essential for GCRs. On the other hand, *rad8-HIRAN* and *rad8-RING* reduced GCR rates, suggesting that Rad8 facilitates GCRs through 3’ DNA-end binding and ubiquitin ligase activity. *rad8-HIRAN* and *rad8-RING* exhibited slightly more pronounced effects on GCR rates than *rad8Δ* (Figs 1B and 2B). In addition to Rad8-dependent GCR events, the mutant proteins may interfere with the GCR events that occur when Rad8 proteins were absent.

### Involvement of the Mms2-Ubc4 ubiquitin-conjugating complex and PCNA lysine 107 (K107) in the Rad8-dependent GCR pathway

**Fig 3:**
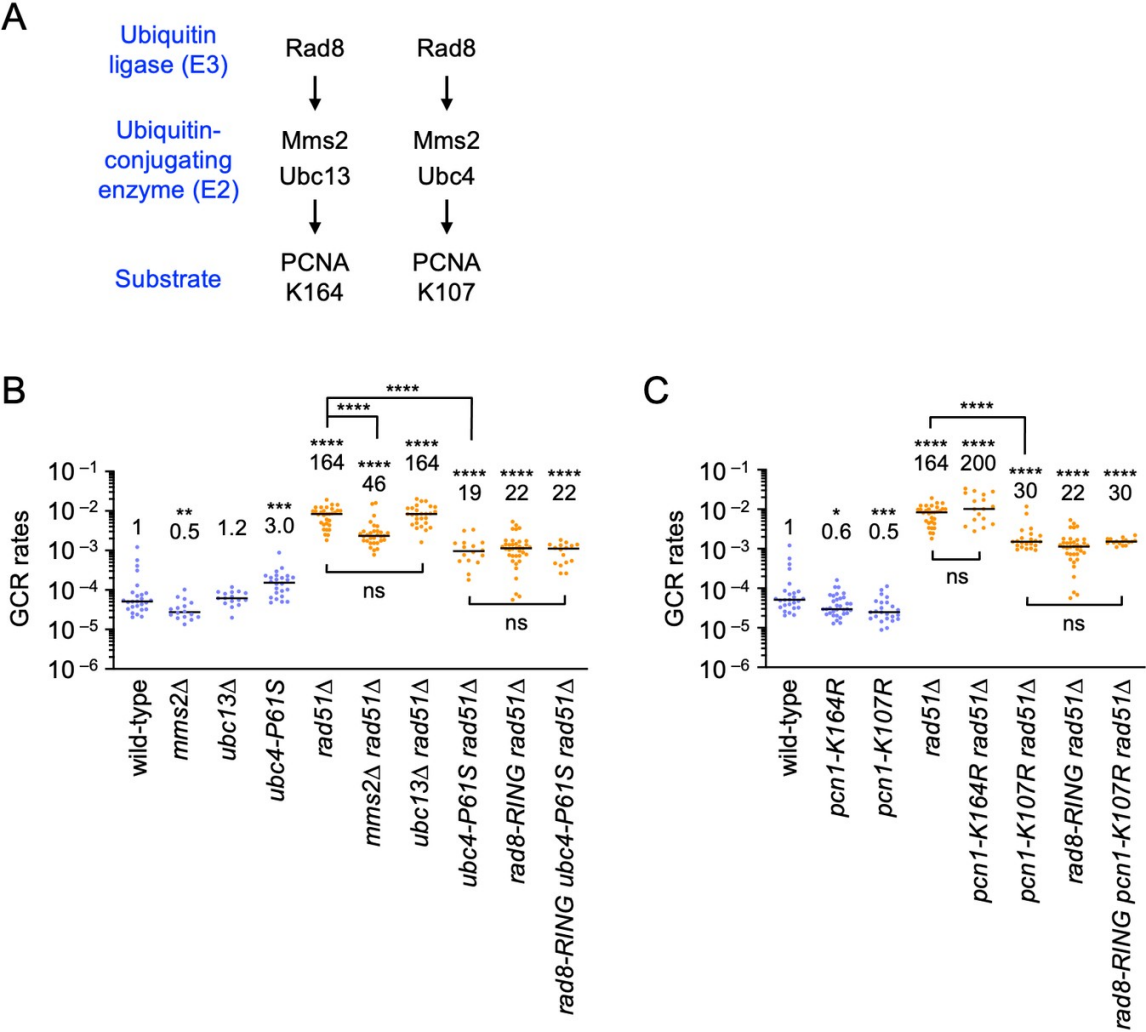
Involvement of the Mms2-Ubc4 ubiquitin-conjugating complex and PCNA K107 in Rad8-dependent GCRs. (A) Two distinct PCNA ubiquitination pathways. Human HLTF, budding yeast Rad5, and fission yeast Rad8 ubiquitinate PCNA K164 with the aid of Mms2-Ubc13 complex. Rad5 ubiquitinates PCNA K107 with the aid of the Mms2-Ubc4 complex. (B) GCR rates of the wild-type, *mms2Δ*, *ubc13Δ*, *ubc4-P61S*, *rad51Δ*, *mms2Δ rad51Δ*, *ubc13Δ rad51Δ*, *ubc4*-*P61S rad51Δ*, *rad8-RING rad51Δ*, and *rad8-RING ubc4-P61S rad51Δ* strains (TNF5369, 6751, 5915, 7484, 5411, 6771, 6115, 7503, 6219, and 7501, respectively). (C) GCR rates of the wild-type, *pcn1-K164R*, *pcn1-K107R*, *rad51Δ*, *pcn1-K164R rad51Δ*, *pcn1-K107R rad51Δ*, *rad8-RING rad51Δ*, and *rad8-RING pcn1-K107R rad51Δ* strains (TNF5369, 6078, 6738, 5411, 6104, 6761, 6219, and 6999, respectively). The two-tailed Mann-Whitney test. Non-significant (ns) *P* > 0.05; * *P*  < 0.05; ** *P*  < 0.01; *** *P* < 0.001; **** *P* < 0.0001. Numerical source data underlying the graphs shown in (B) and (C) are provided respectively in Tables D and E in S1 File.

Rad8 ubiquitinates PCNA at K164 with the aid of Mms2-Ubc13 ubiquitin-conjugating complex, to promote template switching [39] (Fig 3A). Rad8 also ubiquitinates PCNA at K107 with the aid of the Mms2-Ubc4 complex, by inference from the studies of budding yeast Rad5 [44,45]. To determine whether Rad8 facilitates GCRs with these ubiquitin-conjugating enzymes, we constructed *mms2*, *ubc13*, and *ubc4* mutant strains. As *ubc4* is an essential gene, we created the *ubc4-P61S* point mutation that impairs protein ubiquitination [60]. In the wild-type background, *mms2Δ* slightly reduced GCRs whereas *ubc4-P61S* slightly increased GCRs (Fig 3B). It is possible that, independently of Mms2, Ubc4 suppresses GCRs by regulating the Rad51 function [61]. However, in the *rad51Δ* background, both *mms2Δ* and *ubc4-P61S* reduced GCRs, suggesting that the Mms2-Ubc4 complex facilitates GCRs. Either in the presence or absence of Rad51, *ubc13Δ* did not significantly affect GCRs (Figs 3B and S2), showing that the Mms2-Ubc13 complex is not essential for GCRs. It should be noted that the *rad8-RING* mutation did not further reduce GCRs in *ubc4-P61S rad51Δ* cells. These results show that Rad8 facilitates GCRs with the aid of Mms2-Ubc4 rather than Mms2-Ubc13.

The data presented above suggest that the ubiquitination of PCNA K107 rather than the well-known K164 is involved in GCRs (Fig 3A). To test this, we replaced the lysine (K) residue with arginine (R), to which no ubiquitins are conjugated, and determined GCR rates of the *pcn1* mutant strains (Fig 3C, the *pcn1* gene encodes PCNA). In wild-type cells, both *pcn1-K107R* and *pcn1-K164R* slightly reduced GCR rates (see Discussion). However, only *pcn1-K107R* reduced GCRs in *rad51Δ* cells (Figs 3C and S2). Like *pcn1-K107R*, *pcn1-K107A* reduced GCRs (S3 Fig), demonstrating the importance of the ubiquitin acceptor lysine in GCRs. Notably, the *rad8-RING* mutation did not further reduce GCRs in *pcn1-K107R rad51Δ* cells. Together, these results suggest that, with the aid of the Mms2-Ubc4 ubiquitin-conjugating complex, Rad8 ubiquitin ligase ubiquitinates PCNA K107 to facilitate GCRs.

### Rad8 and PCNA K107 play a role in the Rad52-dependent GCR pathway

**Fig 4:**
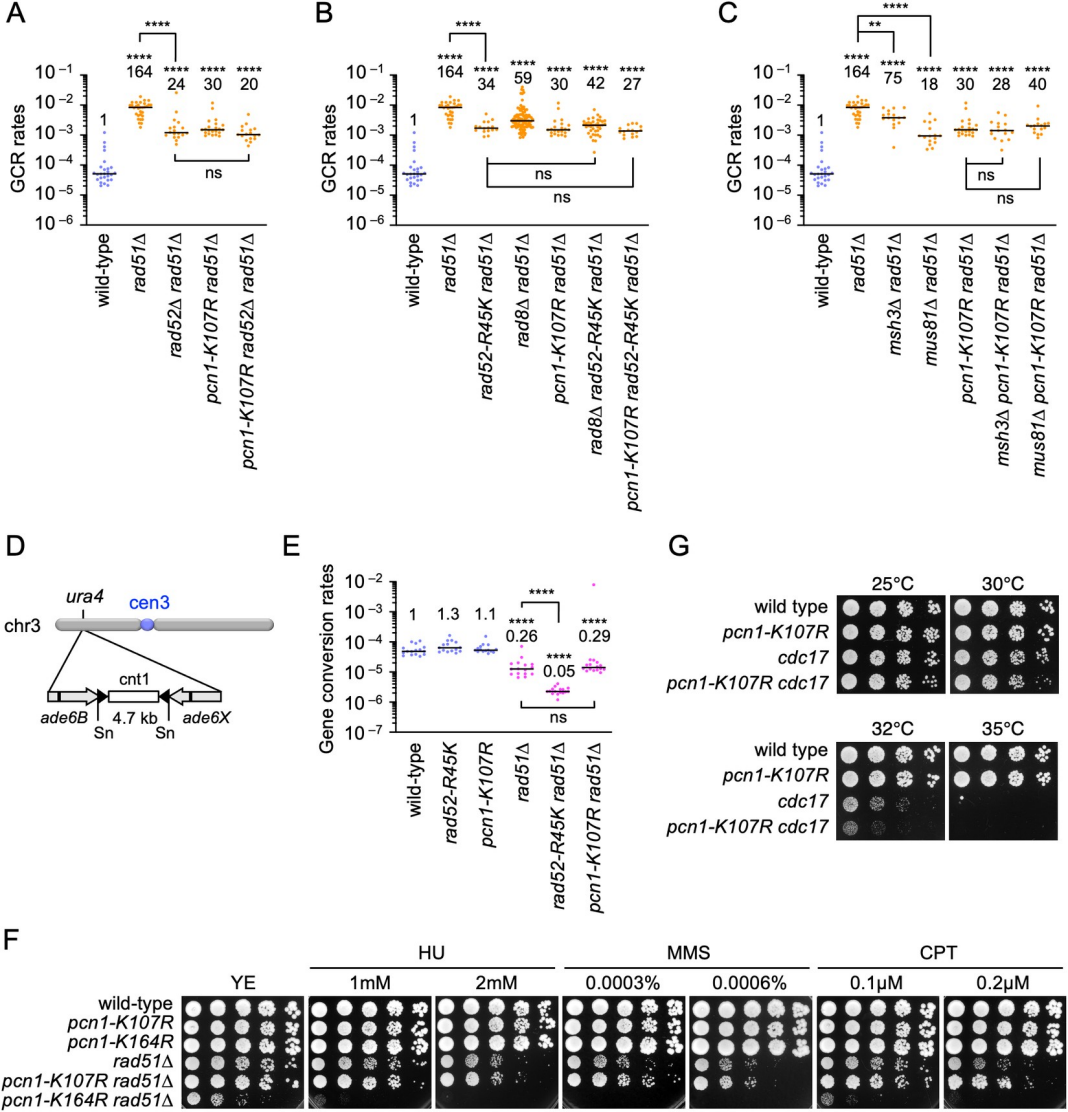
PCNA K107 is involved in the Rad52-dependent GCR pathway. (A) GCR rates of the wild-type, *rad51Δ*, *rad52Δ rad51Δ*, *pcn1-K107R rad51Δ*, and *pcn1-K107R rad52Δ rad51Δ* strains (TNF5369, 5411, 6692, 6761, and 6922, respectively). (B) GCR rates of the wild-type, *rad51Δ*, *rad52-R45K rad51Δ*, *rad8Δ rad51Δ*, *pcn1-K107R rad51Δ*, *rad8Δ rad52-R45K rad51Δ*, and *pcn1-K107R rad52-R45K rad51Δ* strains (TNF5369, 5411, 6616, 5644, 6761, 6704, and 7006, respectively). (C) GCR rates of the wild-type, *rad51Δ*, *msh3Δ rad51Δ*, *mus81Δ rad51Δ*, *pcn1-K107R rad51Δ*, *msh3Δ pcn1-K107R rad51Δ*, and *mus81Δ pcn1-K107R rad51Δ* strains (TNF5369, 5411, 7081, 5974, 6761, 6990, and 7203, respectively). (D) A schematic diagram illustrates the *ade6B* and *ade6X* inverted repeats integrated at the *ura4* locus on the arm region of chr3. Sn, SnaBI. (E) Rates of gene conversion between *ade6B* and *ade6X* heteroalleles in the wild-type, *rad52-R45K*, *pcn1-K107R*, *rad51Δ*, *rad52-R45K rad51Δ*, and *pcn1-K107R rad51Δ* strains (TNF3631, 5995, 7837, 3635, 6021, and 7918 respectively). The two-tailed Mann-Whitney test. Non-significant (ns) *P* > 0.05; ** *P* < 0.01; **** *P* < 0.0001. (F) DNA damage sensitivity. Exponentially growing cells of the wild-type, *pcn1-K107R*, *pcn1-K164R*, *rad51Δ*, *pcn1-K107R rad51Δ*, and *pcn1-K164R rad51Δ* strains (TNF35, 6968, 6948, 2610, 6988, and 6986, respectively) in YE media were five-fold serially diluted with sterilized water and spotted onto YE plates supplemented with HU, MMS, or CPT, at the indicated concentrations. (G) A serial dilution assay to examine temperature sensitive growth. Log-phase cultures of the wild-type, *pcn1-K107R*, *cdc17-K42*, and *pcn1-K107R cdc17-K42* strains (TNF35, 6968, 604, and 6973, respectively) prepared at 25°C in YE media were five-fold serially diluted and spotted onto YE plates. The plates were incubated at the indicated temperatures for 3 to 4 days. Numerical source data underlying the graphs shown in (A-C) and (E) are provided respectively in Tables F, G, H, and I in S1 File.

GCRs occur in Rad52-dependent and Rad52-independent manners in the absence of Rad51 (see Discussion). As previously observed [24], loss of Rad52 only partially reduced GCRs in *rad51Δ* cells (Fig 4A). To determine whether PCNA K107 ubiquitination plays a role in the Rad52-dependent GCR pathway or not, we introduced the *pcn1-K107R* mutation into *rad52Δ rad51Δ* cells and found that *pcn1-K107R* did not significantly reduce GCRs in *rad52Δ rad51Δ* cells (Fig 4A). Like *rad52Δ*, the *rad52-R45K* mutation that impairs SSA activity [24] reduced GCRs in *rad51Δ* cells (*P* = 0.12, the two tailed Mann-Whitney test) (Fig 4B). Neither *rad8Δ* nor *pcn1-K107R* significantly reduced GCRs in *rad52-R45K rad51Δ* cells, suggesting that Rad8-dependent PCNA K107 ubiquitination and Rad52 SSA occur in the same GCR pathway. Msh2-Msh3 MutS-homologs [28] and Mus81 crossover-specific endonuclease [29,30] have been implicated in the Rad52-dependent GCR pathway [11,24]. As previously observed, *msh3Δ* and *mus81Δ* reduced GCRs in *rad51Δ* cells (Fig 4C). However, in *pcn1-K107R rad51Δ* cells, neither *msh3Δ* nor *mus81Δ* significantly reduced GCRs. These results are consistent with the idea that Rad8-dependent PCNA K107 ubiquitination acts in the Rad52-dependent GCR pathway that involves Msh3 and Mus81 endonuclease.

Rad52 promotes gene conversion as well as GCRs in the absence of Rad51 [24]. However, Msh2-Msh3 and Mus81 are not required for gene conversion branch of Rad52-dependent recombination. To determine whether PCNA K107 ubiquitination is also involved in gene conversion, we determined the rate of gene conversion between *ade6B* and *ade6X* heteroalleles that results in adenine prototrophs [27] (Fig 4D and 4E). As previously observed [24], *rad52-R45K* reduced gene conversion only in the absence of Rad51, suggesting the role of Rad52 SSA in Rad51-independent gene conversion. Contrary to *rad52-R45K*, *pcn1-K107R* did not reduce gene conversion even in the absence of Rad51. PCNA K107 was also dispensable for cells to survive exogenous DNA damage (Fig 4F). *pcn1-K107R* did not increase the sensitivity of *rad51Δ* cells to hydroxyurea (HU), methyl methanesulfonate (MMS), and camptothecin (CPT), while *pcn1-K164R* increased the sensitivity [39,48]. Together, these results suggest that, like Msh2-Msh3 and Mus81 [11,24], PCNA K107 ubiquitination is specifically required for nonallelic crossover recombination that results in GCRs but not gene conversion branch of Rad52-dependent recombination, which may play a role in repairing exogenous DNA damage.

In budding yeast, PCNA K107 ubiquitination is induced and required for cell growth in DNA ligase I mutant strains [44]. *pcn1-K107R* also exhibited synthetic growth defects in the fission yeast DNA ligase I *cdc17-K42* mutant [62] at semipermissive temperatures (Fig 4G), suggesting that PCNA K107 ubiquitination occurs when Okazaki fragment maturation fails.

### A PCNA-PCNA interface mutation D150E bypasses the requirement of PCNA K107 and Rad8 ubiquitin ligase for isochromosome formation

**Fig 5:**
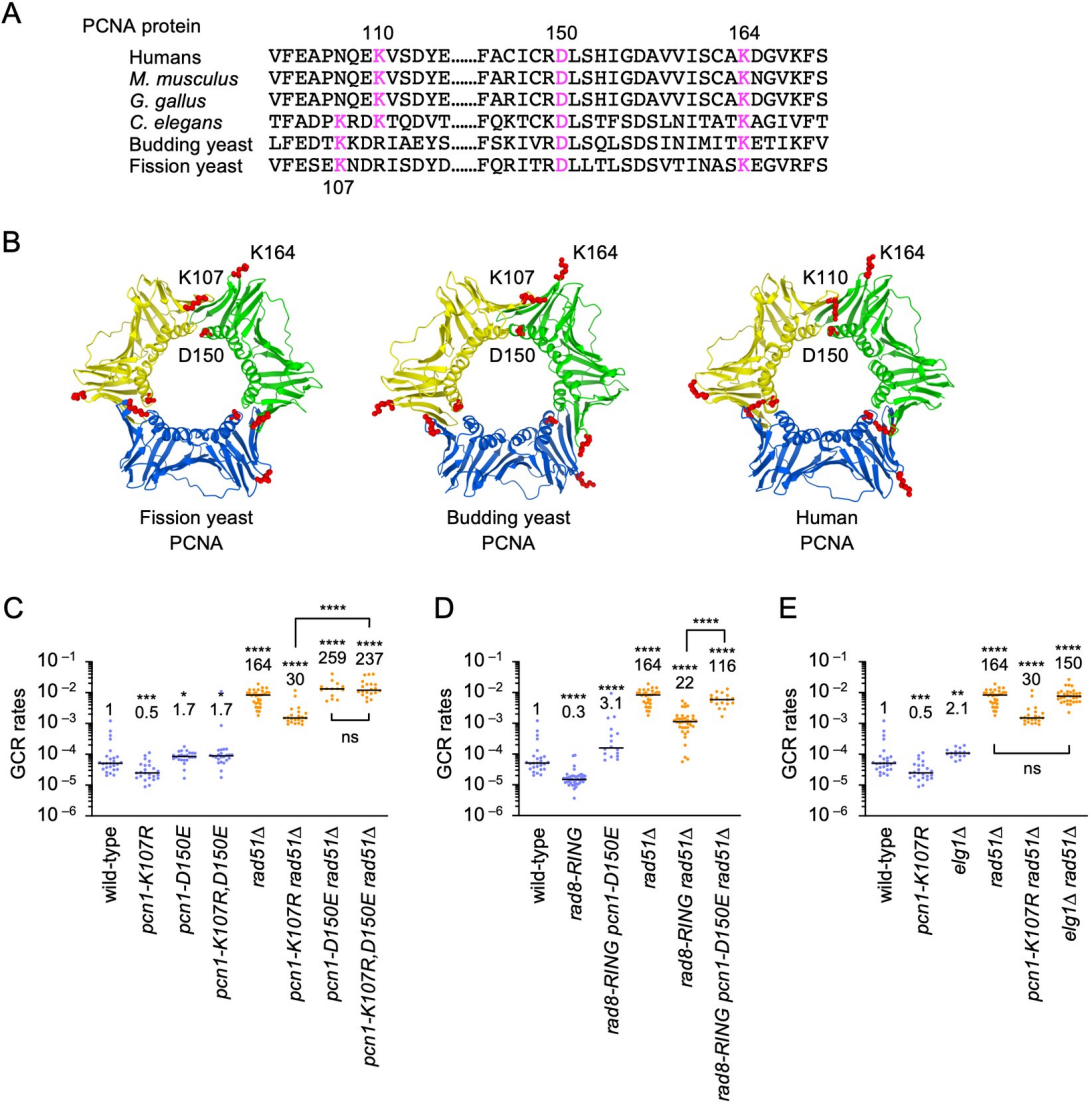
An interface mutation D150E bypasses the requirement of PCNA K107 and Rad8 ubiquitin ligase for GCRs. (A) PCNA amino acid sequences that contain K107, K110, D150, or K164. (B) Structure of fission yeast PCNA was computed by the SWISS-MODEL program using an automated mode [91]. X-ray structure of fission yeast PCNA in a complex with an Spd1 peptide (PDB code 6qh1) was used as the template. X-ray structure of budding yeast PCNA (PDB code 1plr) and human PCNA (PDB code 1vym) are shown. Positions of PCNA K107, K110, D150, and K164 residues (red) were drawn using Mol* Viewer (https://molstar.org/). (C) GCR rates of the wild-type, *pcn1-K107R*, *pcn1-D150E*, *pcn1-K107R*,*D150E*, *rad51Δ*, *pcn1-K107R rad51Δ*, *pcn1-D150E rad51Δ*, and *pcn1-K107R*,*D150E rad51Δ* strains (TNF5369, 6738, 7724, 7727, 5411, 6761, 7744, and 7747, respectively). (D) GCR rates of the wild-type, *rad8-RING*, *rad8-RING pcn1-D150E*, *rad51Δ*, *rad8-RING rad51Δ*, and *rad8-RING pcn1-D150E rad51Δ* strains (TNF5369, 6207, 7750, 5411, 6219, and 7773, respectively). (E) GCR rates of the wild-type, *pcn1-K107R*, *elg1Δ*, *rad51Δ*, *pcn1-K107R rad51Δ*, and *elg1Δ rad51Δ* strains (TNF5369, 6738, 7696, 5411, 6761, and 7741, respectively). The two-tailed Mann-Whitney test. Non-significant (ns) *P* > 0.05; * *P*  < 0.05; ** *P*  < 0.01; *** *P* < 0.001; **** *P* < 0.0001. Numerical source data underlying the graphs shown in (C-E) are provided respectively in Tables J, K, and L in S1 File.

K107 is present in *C*. *elegans*, budding yeast, and fission yeast but not in humans, *M*. *musculus*, or *G*. *gallus* PCNA (Fig 5A). However, instead of K107, K110 is present in humans, *M*. *musculus*, and *G*. *gallus* but not in yeast PCNA. Structural analysis of fission yeast and budding yeast PCNA located K107 but not K164 at the interface between PCNA subunits (Fig 5B), suggesting that the ubiquitination of K107 affects the interaction between PCNA subunits and changes the structure of the PCNA clamp. We reasoned that, if K107 ubiquitination weakens the interaction between PCNA subunits to cause GCRs, a mutation that impairs the interaction will bypass the requirement of K107 ubiquitination for GCRs. D150 is present at the PCNA-PCNA interphase (Fig 5B), and the D150E mutation has been shown to destabilize PCNA homotrimers [46,47]. Introduction of D150E into wild-type or *rad51Δ* cells resulted in a small increase in GCR rates (Fig 5C). However, D150E dramatically increased GCR rates in *pcn1-K107R rad51Δ* cells to the level of *pcn1-D150E rad51Δ*. Most of the GCR products formed in *pcn1-K107R*,*D150E rad51Δ* cells were isochromosomes (S4 Fig), showing that D150E bypassed the requirement of PCNA K107 for homology-mediated GCRs. D150E also bypassed Rad8 ubiquitin ligase requirement for GCRs (Figs 5D and S4). D150E greatly increased GCR rates in *rad8-RING rad51Δ* cells. Together, these data are consistent with the idea that Rad8-dependent PCNA K107 ubiquitination weakens the interaction between PCNA subunits to cause GCRs. Elg1 unloads PCNA from chromatin and facilitates recombination around stalled replication forks [34,35,63]. If K107 ubiquitination facilitates PCNA unloading to cause GCRs, loss of Elg1 may also reduce GCR rates. However, unlike PCNA K107R, *elg1Δ* did not significantly reduce GCRs in wild-type and *rad51Δ* cells (Fig 5E), indicating that Elg1-dependent PCNA unloading is not essential for GCRs. These results suggest that K107 ubiquitination induces the structural change of the PCNA clamp, but not unloading, to cause GCRs.

## Discussion

Here, we provided genetic evidence that fission yeast Rad8 facilitates Rad52-dependent GCRs through PCNA K107 ubiquitination. Loss of Rad8 reduced isochromosome formation in *rad51Δ* cells. Mutations in Rad8 HIRAN and RING finger but not Snf2/Swi2 domain reduced GCRs. *mms2* and *ubc4* but not *ubc13* mutations; PCNA K107R but not K164R reduced GCRs in *rad51Δ* cells. The epistatic analysis suggests that PCNA K107 ubiquitination plays a crucial role in the Rad52-dependent GCR pathway that involves Msh2-Msh3 and Mus81. PCNA K107 is located at the interface between PCNA subunits, suggesting that its ubiquitination affects the PCNA-PCNA interaction to cause GCRs. Indeed, an interface mutation D150E bypassed the requirement of PCNA K107 ubiquitination for GCRs. These data suggest that Rad8-dependent K107 ubiquitination changes the structure of the PCNA clamp to facilitate Rad52-dependent GCRs.

**Fig 6:**
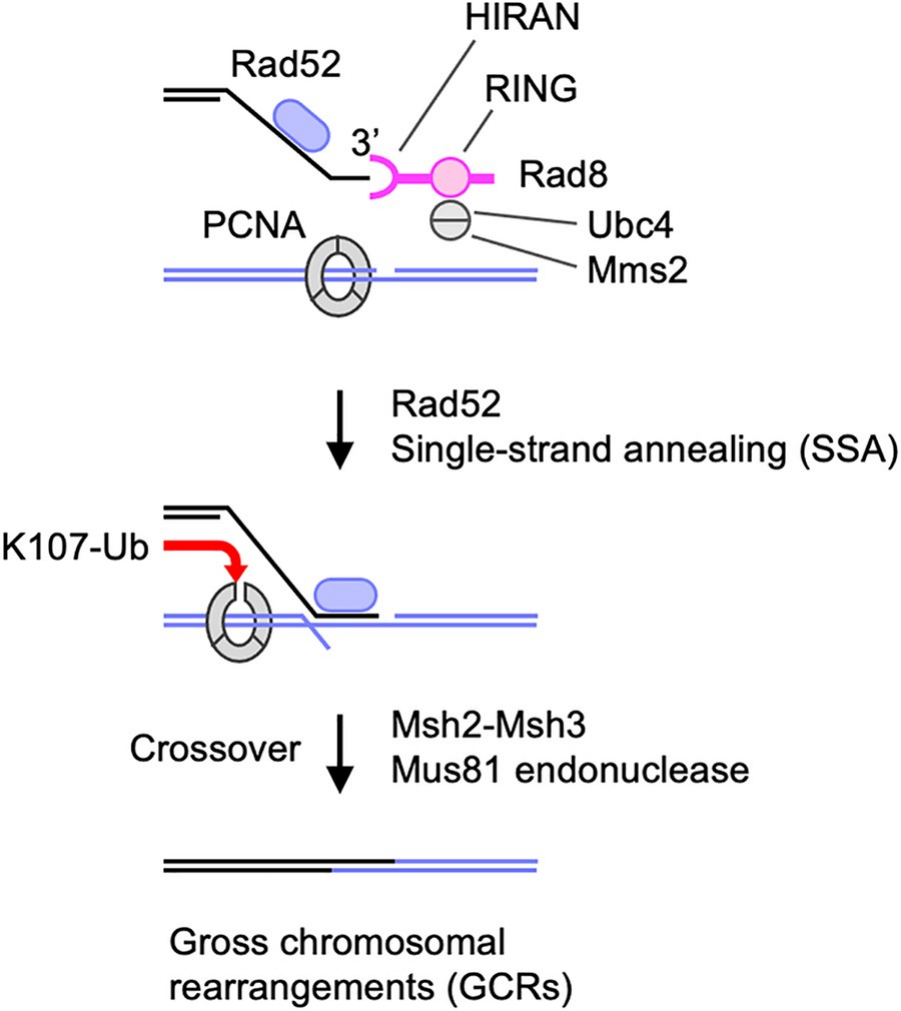
A model explains how Rad8 ubiquitin ligase facilitates GCRs though PCNA K107. Rad8 binds 3’ DNA-ends through HIRAN and interacts with Mms2-Ubc4 E2 complex through RING finger. The Rad8 complex ubiquitinates K107 of PCNA present on template DNA and affects the interaction between PCNA subunits. The structural change of the PCNA clamp facilitates Rad52-dependent recombination and stimulates Msh2-Msh3 MutS-homologs and Mus81 endonuclease. Ub, ubiquitin.

Our data suggest that Rad8 facilitates GCRs through 3’ DNA-end binding and ubiquitin ligase activity (Fig 6). Rad8 ubiquitin ligase acts with Mms2-Ubc13 ubiquitin-conjugating E2 enzymes to cause template switching [39]. However, in the case of GCRs, Rad8 functions with Mms2-Ubc4, as *mms2* and *ubc4* but not *ubc13* mutations reduced GCRs in *rad51Δ* cells. That is further supported by the fact that *rad8-RING* and *ubc4* mutations epistatically reduce GCRs. Ubc4 and Ubc13 contain the cysteine residue critical for the E2 activity, while Mms2 is an E2 variant that lacks the active site [64]. Ubc13 but not Mms2 interacts with budding yeast Rad5 [58]. When Mms2-Ubc4 interacts with Rad8, Ubc4 rather than Mms2 may be the protein that directly binds Rad8 RING finger. E2 complexes determine substrate specificity for Rad8 ubiquitination: Mms2-Ubc4 promotes PCNA K107 while Mms2-Ubc13 causes PCNA K164 ubiquitination [37,44]. It is unknown how Rad8 chooses the E2 partner. The Snf2/Swi2 but not HIRAN domain is essential for PCNA K164 ubiquitination [43,65,66]. In contrast, the HIRAN but not Snf2/Swi2 domain was required for GCRs. It is tempting to suggest that DNA binding via the HIRAN domain facilitates the RING finger to accommodate Mms2-Ubc4 rather than Mms2-Ubc13. However, it is also possible that the *rad8-HIRAN* mutation directly or indirectly interferes with Rad8-PCNA interaction [67] and thereby reduces PCNA K107 ubiquitination and GCRs. It is also possible that DNA binding of the HIRAN domain has a ubiquitination-independent role to cause GCRs [43,68,69]. Further study is required to understand how Rad8 interacts with Mms2-Ubc4 and Mms2-Ubc13 to ubiquitinate different substrates.

In the absence of Rad51, isochromosomes are formed in Rad52-dependent and Rad52-independent manners [24]. Several lines of evidence suggest that Rad8-dependent PCNA K107 ubiquitination plays a crucial role in Rad52-dependent GCRs. Firstly, both Rad8 and Rad52 are involved specifically in homology-mediated GCRs that result in isochromosome formation. In *rad51Δ* cells, loss of Rad8 and Rad52 eliminated 75 and 90% of isochromosomes, respectively, but did not reduce chromosomal truncations [24]. Secondly, *rad52Δ* and PCNA K107R epistatically reduced GCRs. In addition, the *rad52-R45K* mutation that impairs SSA activity [24] epistatically reduced GCRs with *rad8Δ* as well as PCNA K107R. Thirdly, PCNA K107R reduced GCRs epistatically with loss of Msh3 or Mus81, both of which have been implicated in Rad52-dependent GCRs [24]. Collectively, it seems that Msh2-Msh3 MutS-homologs, Mus81 endonuclease, and Rad8-dependent PCNA K107 ubiquitination are involved in Rad52-dependent GCR pathway. In addition to GCRs, Rad52 facilitates gene conversion between inverted repeats in the absence of Rad51. However, like Msh2-Msh3 and Mus81, PCNA K107 was dispensable for the gene conversion in *rad51Δ* cells, suggesting that PCNA K107 ubiquitination is specifically involved in the GCR branch of Rad52-dependent recombination. Interestingly, human K110 is also located at the PCNA-PCNA interface (Fig 5B), and its ubiquitination has been detected in cultured cells [70], suggesting that human K110 is the counterpart of yeast K107.

Homology-mediated GCRs occur even in the absence of Rad52. Isochromosome formation is increased in *rad52Δ rad51Δ* and *rad8Δ rad51Δ* cells compared to wild-type cells ([24] and this study). The fission yeast Fbh1 helicase disrupts Rad51 nucleoprotein filaments [61], and a mutation in Fbh1 suppresses *rad52Δ* mutant phenotypes [30,71–73], raising the possibility that a residual level of GCRs in *rad52Δ rad51Δ* cells is caused by a spontaneous mutation in the *fbh1* gene. However, that is unlikely, because Rad51 is required for the *fbh1* suppression [71]. Indeed, DNA sequencing showed no *fbh1* mutations in the parental cells of the *rad52* strains used in this study (S2 File) and in two independent GCR clones of the *rad52Δ rad51Δ* strain (S3 File). Recombination between centromere inverted repeats may occur even in the absence of Rad52 and Rad51, resulting in isochromosome formation. Interestingly, in budding yeast, most of the heteroallelic recombination in *rad52Δ rad51Δ* cells are crossover recombination while noncrossover recombination in wild-type cells [74,75]. Rad51 and Rad52-independent recombination has been reported in other repetitive regions of chromosomes. In budding yeast, Rad51 and Rad52 are dispensable for repairing DSBs formed at the replication fork barrier site in rDNA repeats in wild type [76]. Alternative lengthening of telomeres (ALT) is a telomerase-independent but recombination-dependent process that maintains telomeres. Rad51 is dispensable for ALT in human cells, and there are Rad52-dependent and Rad52-independent ALT pathways [77]. POLD3 and POLD4 subunits of DNA polymerases δ (Pol δ) are required for ALT in Rad52-knockout cell lines [77], suggesting the role of Pol δ in Rad52-independent recombination. Interestingly, budding yeast Pol δ is also required for microhomology/microsatellite-induced replication (MMIR) or microhomology-mediated repair (MHMR) that occurs in the absence of Rad52 [78,79], suggesting the role of Pol δ in stabilizing otherwise unstable joint molecules. Break-induced replication [80] and DSB-independent replication template exchange [6,81] also require Pol δ. Although a mutation in a Cdc27/Pol32/POLD3 subunit of Pol δ did not reduce GCRs in *rad51Δ* cells [11], Pol δ could be required for isochromosome formation in *rad51Δ rad52Δ* cells [82]. One of the future directions is to understand how homology-mediated GCRs occur independently of Rad52.

Post-translational modifications of PCNA at K164 affect protein interaction. K164 ubiquitination facilitates PCNA binding to translesion synthesis polymerases and Mgs1/ZRANB3 helicase [36]. In budding yeast, Small ubiquitin-like modifier (SUMO) protein is also attached to PCNA K164. K164 SUMOylation causes PCNA interaction with Srs2 helicase that suppresses Rad51-dependent recombination by dissociating Rad51 protein from ssDNA [83]. However, the C-terminal domain of budding yeast Srs2 that interacts with SUMOylated PCNA is not conserved in fission yeast Srs2 [39]. Nonetheless, loss of Rad51 resulted in slow growth phenotypes and increased sensitivities to DNA damaging agents in *pcn1-K164R* but not in *pcn1-K107R* cells [39] (Fig 4F). PCNA K164R slightly but significantly reduced GCRs in wild-type but not in *rad51Δ* cells (Fig 3C). PCNA K164R may channel DNA repair into Rad51-dependent gene conversion pathway.

How does PCNA K107 ubiquitination cause GCRs? Our data suggest that K107 ubiquitination changes the PCNA clamp structure to facilitate GCRs (Fig 6). PCNA subunits interact with each other to form ring-shaped homotrimers. K107 is located at the interface between PCNA subunits. An interface mutation D150E [46,47] bypassed the requirement of Rad8 RING finger and PCNA K107 for GCRs, showing that the ubiquitin or ubiquitin chain at K107 is not essential for GCRs and that weakening the PCNA-PCNA interaction is sufficient to cause GCRs. PCNA K107R mutation did not significantly change the level of Rad52 foci accumulated in the absence of Rad51 [84] (S5 Fig), suggesting that PCNA K107 ubiquitination is not required for Rad52 to localize to damage sites. However, the PCNA clamp present at the end of Okazaki fragments can be a structural hinderance of Rad52-dependent SSA. Consistent with this idea that PCNA K107 ubiquitination occurs when PCNA is bound to Okazaki fragments, PCNA K107R showed synthetic growth defects in DNA ligase I *cdc17* mutant cells. In budding yeast, PCNA K107R also causes growth defects in DNA ligase I mutants, and K107 ubiquitination facilitates checkpoint activation that depends on the assembly of checkpoint proteins onto ssDNA [44,45]. RFC-like complexes containing Elg1 unload PCNA from DNA after Okazaki fragment ligation [34,35]. Elg1 promotes Rad51 and Rad52 proteins’ recruitment to stalled replication forks and facilitates nearby recombination [63], suggesting that PCNA unloading facilitates recombination around stalled forks. An interface mutation D150E also bypasses the role of Elg1 in PCNA unloading and promoting recombination around stalled replication forks [46,63]. However, unlike PCNA K107R, loss of Elg1 did not reduce GCRs, suggesting that Elg1-dependent PCNA unloading is not required to cause GCRs. However, we do not exclude the possibility that K107 ubiquitination facilitates PCNA unloading, by itself or with the factors other than Elg1. Like Msh2-Msh3 MutS-homologs and Mus81 endonuclease [24], it appears that PCNA K107 ubiquitination is specifically required for the GCR branch of Rad52-dependent recombination. It has been reported that the PCNA clamp interacts with Msh2-Msh3 [85] and Mus81 to enhance endonuclease activity [86]. The structural change of the PCNA clamp induced by K107 ubiquitination might facilitate the interaction with Msh2-Msh3 and Mus81 and result in GCRs (Fig 6). Our Western blot failed to detect PCNA ubiquitination that clearly depends on K107, suggesting that K107 ubiquitination occurs in a limited fraction of PCNA molecules (S6 Fig). In the future, biochemical study is required to detect PCNA K107 ubiquitination and address the effect of K107 ubiquitination on the structure and function of PCNA.

This study has uncovered the role of Rad8 ubiquitin ligase and PCNA K107 in Rad52-dependent GCRs. We also suggest that human PCNA K110 is the counterpart of yeast PCNA K107. Interestingly, HLTF, the human homolog of Rad8, is often amplified and overexpressed in cancer [68], suggesting that HLTF causes GCRs and facilitates tumorigenesis.

## Materials and methods

### Genetic procedures and yeast strains

The fission yeast strains used in this study are listed in S1 Table. Standard genetic procedures and fission yeast media were used as previously described [11]. Pombe minimal glutamate (PMG) medium is identical to Edinburgh minimal medium (EMM), except containing 3.75 g/l monosodium L-glutamate instead of 5 g/l ammonium chloride. Yeast nitrogen base (YNB) medium contained 1.7 g/l yeast nitrogen base (BD Biosciences, San Jose, California, Difco 233520), 5 g/l ammonium sulphate (Nacalai Tesque, Kyoto, Japan, 02619–15), and 2% glucose. YE, YNB, EMM, and PMG contain 225 mg/l of each amino acid when indicated. FOA+UA is a YNB derivative supplemented with 56 mg/l uracil, 225 mg/l adenine, and 1 g/l 5-fluoroorotic acid monohydrate (Apollo Scientific, Stockport, United Kingdom, PC4054). Yeast cells were grown at 30°C. When we inoculated liquid media using slow-growing mutant strains, we did not take exceptionally large colonies, if any, because they might contain a mutation that suppresses the growth and other defects. S7 Fig shows a picture of *rad52Δ rad51Δ* colonies formed on EMM+UA plates.

The *rad8-HIRAN* mutant strain was created by the pop-in/pop-out gene replacement [87]. pTN1192 plasmid containing *ura4*^+^ and *rad8-HIRAN* was linearised by BglII digestion at a unique site in the *rad8* region and introduced into *ura4-D18* cells. Ura^+^ transformants were selected on EMM. After confirmation of the correct integration by PCR and DNA sequencing, the *rad8*:*ura4*^+^:*rad8-HIRAN* strain was grown in YE media supplemented with uracil (YE+U) and then plated on FOA+U media to select Ura^−^ colonies, resulting from “pop-out” of the *ura4*^+^ marker. DNA sequencing confirmed the retention of the *rad8*-*HIRAN* mutation in the Ura^−^ cells. *rad8-ATPase* and *rad8-RING* mutant strains were created essentially in the same way, but BglII-digested pTN1191 plasmid containing *rad8-ATPase* and PacI-digested pTN1193 plasmid containing *rad8-RING* were used, respectively.

The *pcn1-K107R* mutant strain was constructed as follows. First, we created the DNA fragment containing the *pcn1-K107R* mutation. Two partially overlapping fragments were amplified separately from yeast genomic DNA: a 0.6 kb fragment using pcn1-F3 and pcn1-K107RB primers, and a 1.4 kb fragment using pcn1-K107RT and pcn1-R2 primers. pcn1-K107RB and pcn1-K107RT contain the *pcn1-K107R* mutation. The two PCR products were mixed and connected by the second round of PCR in the presence of pcn1-F3 and pcn1-R2 primers, resulting in the formation of a 1.9 kb fragment containing the *pcn1-K107R* mutation. To create the *pcn1-K107R* strain, we first introduced the *ura4*^+^ gene 0.7 kb downstream of the *pcn1*^+^ gene in *ura4-D18* cells, making *pcn1*^+^:*ura4*^+^ cells. Then, the 1.9 kb PCR fragment that contains the *pcn1-K107R* mutation and encompasses the *ura4*^+^ integration site was introduced into *pcn1*^+^:*ura4*^+^ cells. Ura^−^ transformants were selected on FOA+U plates. DNA sequencing confirmed the correct integration of *pcn1-K107R*. *pcn1-K107A*, *pcn1-K164R*, and *pcn1-D150E* strains were created essentially in the same way, except that pcn1-K107AB/pcn1-K107AT, pcn1-K164RB/pcn1-K164RT, and pcn1-D150EB/pcn1-D150ET primers, instead of pcn1-K107RB/pcn1-K107RT, were used to create *pcn1-K107A*, *pcn1-K164R*, and *pcn1-D150E* mutations, respectively. The *pcn1-K107R*,*D150E* double mutation was created using genomic DNA of *pcn1-K107R* cells and pcn1-D150EB/pcn1-D150ET primers.

We created the *ubc4-P61S* mutation as follows. Two partially overlapping fragments were amplified separately: a 0.7 kb fragment using ubc4-F1 and ubc4-P61S-B primers, and a 1.5 kb fragment using ubc4-P61S-T and ubc4-R4 primers. ubc4-P61S-B and ubc4-P61S-T contain the *ubc4-P61S* mutation. The two PCR products were mixed and connected by the second round of PCR in the presence of ubc4-F1 and ubc4-R4 primers, resulting in the formation of a 2.2 kb fragment containing the *ubc4-P61S* mutation. To create the *ubc4-P61S* strain, we first introduced the *ura4*^+^ gene 0.6 kb downstream of the *ubc4*^+^ gene in *ura4-D18* cells, making *ubc4*^+^:*ura4*^+^ cells. Then, the 2.2 kb PCR fragment that contains the *ubc4-P61S* mutation and encompasses the *ura4*^+^ integration site was introduced into *ubc4*^+^:*ura4*^+^ cells. Ura^−^ transformants were selected on FOA+U plates. DNA sequencing confirmed the correct integration of *ubc4-P61S*. Sequences of PCR primers used are listed in S2 Table.

### Plasmids

We constructed the plasmid pTN1192 containing *ura4*^+^ and *rad8-HIRAN* as follows. Two partially overlapping fragments were amplified separately from yeast genomic DNA: a 1.4 kb fragment using rad8-KpnI and rad8-HIRAN-B primers, and a 1.0 kb fragment using rad8-HIRAN-T and rad8-SalI primers. rad8-HIRAN-B and rad8-HIRAN-T contain the *rad8-HIRAN* mutation. The two PCR products were mixed and connected by the second round of PCR in the presence of rad8-KpnI and Rad8-SalI primers. A 2.3 kb KpnI-SalI restriction fragment of the PCR product containing the *rad8-HIRAN* mutation was introduced between KpnI-SalI sites of pTN782 [88], which contains a 1.5 kb HindIII-SspI fragment containing the *ura4*^+^ gene between HindIII-EcoRV sites of pBluescript II KS^+^ (Agilent, Santa Clara, California).

The plasmid pTN1191 containing *ura4*^+^ and *rad8-ATPase*, and the plasmid pTN1193 containing *ura4*^+^ and *rad8-RING*, were created essentially in the same way as described above. To create pTN1191, rad8-SacII/rad8-ATPase-B/rad8-ATPase-T/rad8-BamHI primers were used, and a 2.9 kb SacII-BamHI restriction fragment of the 2nd PCR product was introduced between SacII-BamHI sites of pTN782. To create pTN1193, rad8-1/rad8-RING-B/rad8-RING-T/rad8-EcoRI primers were used, and a 1.1 kb BamHI-EcoRI restriction fragment of the 2nd PCR product was introduced between BamHI-EcoRI sites of pTN782.

### GCR rates

Spontaneous rates of GCRs that result in loss of *ura4*^+^ and *ade6*^+^ markers were determined essentially as previously described [24]. Yeast cells harbouring ChL^C^ were incubated on EMM+UA plates for 6–8 days. 10 ml EMM+UA was inoculated with a single colony from EMM+UA plates and incubated for 1–2 days. Cells were plated on YNB+UA and FOA+UA plates and incubated for 5–8 days. Leu^+^ and Leu^+^ Ura^−^ colonies formed on YNB+UA and FOA+UA plates, respectively, were counted. Leu^+^ Ura^−^ colonies were streaked on EMM+UA plates to examine the colony size and transferred to EMM+U plates to inspect adenine auxotrophy. The number of Leu^+^ Ura^−^ Ade^−^ was obtained by subtracting the number of Leu^+^ Ura^−^ Ade^+^ from that of Leu^+^ Ura^–^. Rates of GCR per cell division were calculated [89], using the numbers of Leu^+^ cells and Leu^+^ Ura^−^ Ade^−^ cells, using Microsoft Excel for Mac 16 (Microsoft, Redmond, Washington). The GCR strains were constructed in the *h- (smt0)* background [90] (S1 Table), in which no DSBs are formed at the *mat1* locus.

### Pulsed-field gel electrophoresis (PFGE) analysis of GCR products

1×10^8^ cells grown at 25°C were collected, suspended in 2.5 ml ice-cold 50 mM EDTA, and stored at 4°C. After centrifugation, cells were suspended in 1 ml CSE buffer (20mM citrate phosphate, 1 M sorbitol, 50 mM EDTA, pH 5.6). After adding 5 to 10 μl Zymolyase 20T (Seikagaku, Tokyo, Japan, 25 mg/ml) and 5 to 10 μl lyzing enzyme (Sigma, St. Louis, Missouri, 25 mg/ml), cell suspensions were incubated for 20 to 50 min at 30°C. After centrifugation at 700 rpm for 10 min at 4°C (TOMY, Tokyo, Japan, MX-201, TMS-21 swing rotor), the pellet was suspended in 140 μl CSE buffer. After adding 140 μl 1.6% low melting agarose gel (Nacalai Tesque, 01161–12) pre-heated at 50°C, the suspension of spheroplasts was transferred into a mould. After 20 min at 4°C, the agarose plugs were incubated in SDS-EDTA solution (1% SDS, 0.25 M EDTA) for 2 h at 60°C. The plugs were transferred into ESP solution (0.5M EDTA, 1% N-lauroylsarcosine, 1.5 mM calcium acetate) supplemented with 0.5 mg/ml proteinase K and incubated overnight at 50°C. The plugs were transferred into another ESP solution supplemented with 0.5 mg/ml proteinase K and incubated at 50°C for an additional 8 h. The plugs were stored in TE buffer (10 mM Tris-HCl (pH8.0), 1 mM EDTA) at 4°C. Chromosomal DNAs prepared in agarose plugs were resolved using a CHEF-DRII pulsed-field electrophoresis system (Bio-Rad, Hercules, California). Broad-range PFGE ran at 2 V/cm with a pulse time of 1500 s or 1600 s for 42 h followed by 2.4 V/cm with a pulse time of 180 s for 4 h, at 4°C in 1×TAE buffer (40 mM Tris-acetate, 1 mM EDTA) using 0.55% Certified Megabase agarose gel (Bio-Rad, 161–3109). Short-range PFGE ran at 4.2 or 4.5 V/cm with a pulse time from 40 to 70 s for 24 h, at 4°C in 0.5×TBE buffer (89 mM Tris-borate, 2 mM EDTA) using 0.55% Certified Megabase agarose gel, otherwise indicated. DNA was stained with 0.2 μg/ml ethidium bromide (EtBr) (Nacalai Tesque, 14631–94) and detected using a Typhoon FLA9000 gel imaging scanner (GE Healthcare, Chicago, Illinois). Gel images were processed using ImageJ 1.8.0 (NIH, United States).

### Gene conversion rates

Spontaneous rates of gene conversion that occurs between *ade6B* and *ade6X* heteroalleles integrated at the *ura4* locus [27] were determined. 10 ml PMG+UA was inoculated with a single colony from PMG+UA plates and incubated for 1–2 days. Cells were plated onto PMG+UA and PMG+U media. After 4–7 days’ incubation of the plates, colonies formed on PMG+UA and PMG+U were counted to determine the number of colony-forming units and Ade^+^ prototrophs, respectively. The rates of gene conversion per cell division were calculated [89], using Microsoft Excel for Mac 16.

### Cell imaging

Exponentially growing cells in EMM were collected, seeded on glass-bottom dishes (Matsunami Glass, Osaka, Japan, D11130H), and observed using a DeltaVision Personal fluorescence microscopy system (GE Healthcare), which is based on an Olympus wide-field IX71 fluorescence microscope equipped with a CoolSNAP HQ2 CCD camera (Photometrics, Tucson, Arizona) and an oil-immersion objective lens (UAPO 40×; NA = 1.35; Olympus, Tokyo, Japan).

### Western blotting

Western blotting was performed as described previously [88]. 1×10^8^ cells from log-phase cultures in YE media were collected. Cells were suspended in 150 μl of 10% trichloroacetic acid (TCA) and disrupted by a bead neater (TOMY, MS-100) in the presence of acid-washed glass beads. After adding 250 μl of 5% TCA, cell extracts were kept on ice for 30 min. The pellet recovered after centrifugation at 5,000 rpm for 10 min at 4°C (TOMY, Kitman) was suspended in 200 μl of SDS-elution buffer (0.5 M Tris base, 28.125 mM Tris-HCl (pH6.8), 11.25% glycerol, 0.9% sodium dodecyl sulfate, 5% β-mercaptoethanol, 0.0045% bromophenol blue) and incubated at 95°C for 2 min. The supernatant was recovered after centrifugation at 12,000 rpm for 10 min at room temperature. The cell extracts were separated by sodium dodecyl sulfate-polyacrylamide gel electrophoresis (SDS-PAGE) (acrylamide to bisacrylamide ratio, 37.5:1) and transferred onto PolyScreen PVDF Transfer Membrane (Perkin Elmer, NEF1002001PK). Anti-PCNA antibodies (1:2,000) and Peroxidase AffiniPure HRP-conjugated goat anti-rabbit IgG (Jackson ImmunoResearch Laboratories, 111-035-003; 1:10,000) were used as the primary and secondary antibodies, respectively. The blots were developed using Supersignal West Femto substrate (ThermoScientific, 34095). Images were acquired using ImageQuant LAS 500 (GE Healthcare).

### Statistical analyses

Two-tailed Mann-Whitney tests and Fisher’s exact tests were performed using GraphPad Prism for Mac version 8 (GraphPad Software, San Diego, California). Two-tailed student’s *t*-tests were performed using Microsoft Excel for Mac 16.

## Supporting information

S1 Table Fission yeast strains used in this study. (XLSX)

S2 Table Oligonucleotide sequences. (XLSX)

S1 Fig GCR products formed in wild-type, *rad8Δ*, *rad51Δ*, and *rad8Δ rad51Δ* cells. Chromosomal DNAs prepared from the parental and independent GCR clones of the wild-type, *rad8Δ*, *rad51Δ*, and *rad8Δ rad51Δ* strains (TNF5369, 5549, 5411, and 5644, respectively) were separated by broad- and short-range PFGE and stained with EtBr. Sample number of translocations and truncations are highlighted in blue and red, respectively.
(TIF)

S2 Fig GCR products formed in *ubc13Δ rad51Δ* and *pcn1-K164R rad51Δ* cells. Chromosomal DNAs prepared from the parental and independent GCR clones of the *ubc13Δ rad51Δ* and *pcn1-K164R rad51Δ* strains (TNF6115 and 6104, respectively) were separated by broad- and short-range PFGE and stained with EtBr. Short-range PFGE ran at 4.5 V/cm with a pulse time from 4 to 120 s for 48 h at 4°C.
(TIF)

S3 Fig The *pcn1-K107A* mutation reduces GCR rates. GCR rates of the wild-type, *pcn1-K107A*, *rad51Δ*, and *pcn1-K107A rad51Δ* strains (TNF5369, 6699, 5411, and 6719, respectively). The two-tailed Mann-Whitney test. ** *P*  < 0.01; **** *P* < 0.0001. Numerical source data underlying the graph are provided in Table M in S1 File.
(TIF)

S4 Fig GCR products formed in *pcn1-K107R*,*D150E rad51Δ* and *rad8-RING pcn1-D150E rad51Δ* cells. Chromosomal DNAs prepared from the *pcn1-K107R*,*D150E rad51Δ* and *rad8-RING pcn1-D150E rad51Δ* strains (TNF7747 and 7773, respectively) were separated by broad- and short-range PFGE and stained with EtBr.
(TIF)

S5 Fig PCNA K107 is not essential for Rad52 focus formation. (A) Rad52-mCherry foci were observed by fluorescence microscopy. DIC, differential interference contrast. A scale bar indicates 5 μm. (B) Percentages of cells containing Rad52 foci in the wild-type, *pcn1-K107R*, *rad51Δ*, and *pcn1-K107R rad51Δ* strains (TNF4462, 7387, 7800, and 7802, respectively). Bars represent the mean of three independent experiments shown as dots. > 200 cells were counted in each experiment. The two-tailed student’s *t*-test. Non-significant (ns) *P* > 0.05; **** *P* < 0.0001. Numerical source data underlying the graph shown in (B) are provided in Table N in S1 File.
(TIF)

S6 Fig Detection of PCNA by Western blotting. Extracts prepared from the wild-type, *pcn1-K107R*, *pcn1-K164R*, *pcn1-K107R*,*K164R*, *rad51Δ*, *pcn1-K107R rad51Δ*, *pcn1-K164R rad51Δ*, and *pcn1-K107R*,*K164R rad51Δ* strains (TNF35, 6968, 6948, 6996, 2610, 6988, 6986, and 7012, respectively) were resolved by 10% SDS-PAGE. PCNA was detected using anti-PCNA antibodies at 1:2,000 dilution. Sizes of pre-stained protein markers (Nacalai tesque, 02525–35) are indicated on the right of the image. It might be worth noting that the *pcn1-K107R* mutation increased the mobility of PCNA in SDS-PAGE.
(TIF)

S7 Fig Yeast colonies on EMM+UA plates. The *rad52Δ rad51Δ* and *pcn1-K107R rad52Δ rad51Δ* strains (TNF6692 and 6922, respectively) were incubated on EMM+UA plates. We took a picture of colonies after seven days’ incubation at 30°C. There were no exceptionally large colonies on this plate.
(TIF)

S1 File Table A: Numeric source data underlying the graph shown in Fig 1B. Table B: Numeric source data underlying the graph shown in Fig 1D. Table C: Numeric source data underlying the graph shown in Fig 2B. Table D: Numeric source data underlying the graph shown in Fig 3B. Table E: Numeric source data underlying the graph shown in Fig 3C. Table F: Numeric source data underlying the graph shown in Fig 4A. Table G: Numeric source data underlying the graph shown in Fig 4B. Table H: Numeric source data underlying the graph shown in Fig 4C. Table I: Numeric source data underlying the graph shown in Fig 4E. Table J: Numeric source data underlying the graph shown in Fig 5C. Table K: Numeric source data underlying the graph shown in Fig 5D. Table L: Numeric source data underlying the graph shown in Fig 5E. Table M: Numeric source data underlying the graph shown in S3 Fig. Table N: Numeric source data underlying the graph shown in S5B Fig.
(XLSX)

S2 File DNA sequencing data of the *fbh1* gene in the *rad52* mutant strains used in this study. Genomic DNA was extracted from *rad52Δ rad51Δ*, *pcn1-K107R rad52Δ rad51Δ*, *rad52-R45K rad51Δ*, *rad8Δ rad52-R45K rad51Δ*, *pcn1-K107R rad52-R45K rad51Δ*, *rad52-R45K*, and *rad52-R45K rad51Δ* cells (TNF6692, 6922, 6616, 6704, 7006, 5995, and 6021) before selecting GCR or gene conversion clones. Nucleotide sequences of the primers used in DNA sequencing are shown in S2 Table. (AB1 and DNA).
(ZIP)

S3 File DNA sequencing data of the *fbh1* gene in GCR clones of the *rad52Δ rad51Δ* strain. Genomic DNA was extracted from two independent GCR clones of the *rad52Δ rad51Δ* strain (TNF6692). Nucleotide sequences of the primers used in DNA sequencing are shown in S2 Table. (AB1 and DNA).
(ZIP)
