# Supplementary figures and images for: Fission yeast Rad8/HLTF facilitates Rad52-dependent chromosomal rearrangements through PCNA lysine 107 ubiquitination
Source: PLoS Genet. 2021 Jul 22;17(7):e1009671. doi: 10.1371/journal.pgen.1009671 (PMC8297803; doi:10.1371/journal.pgen.1009671)

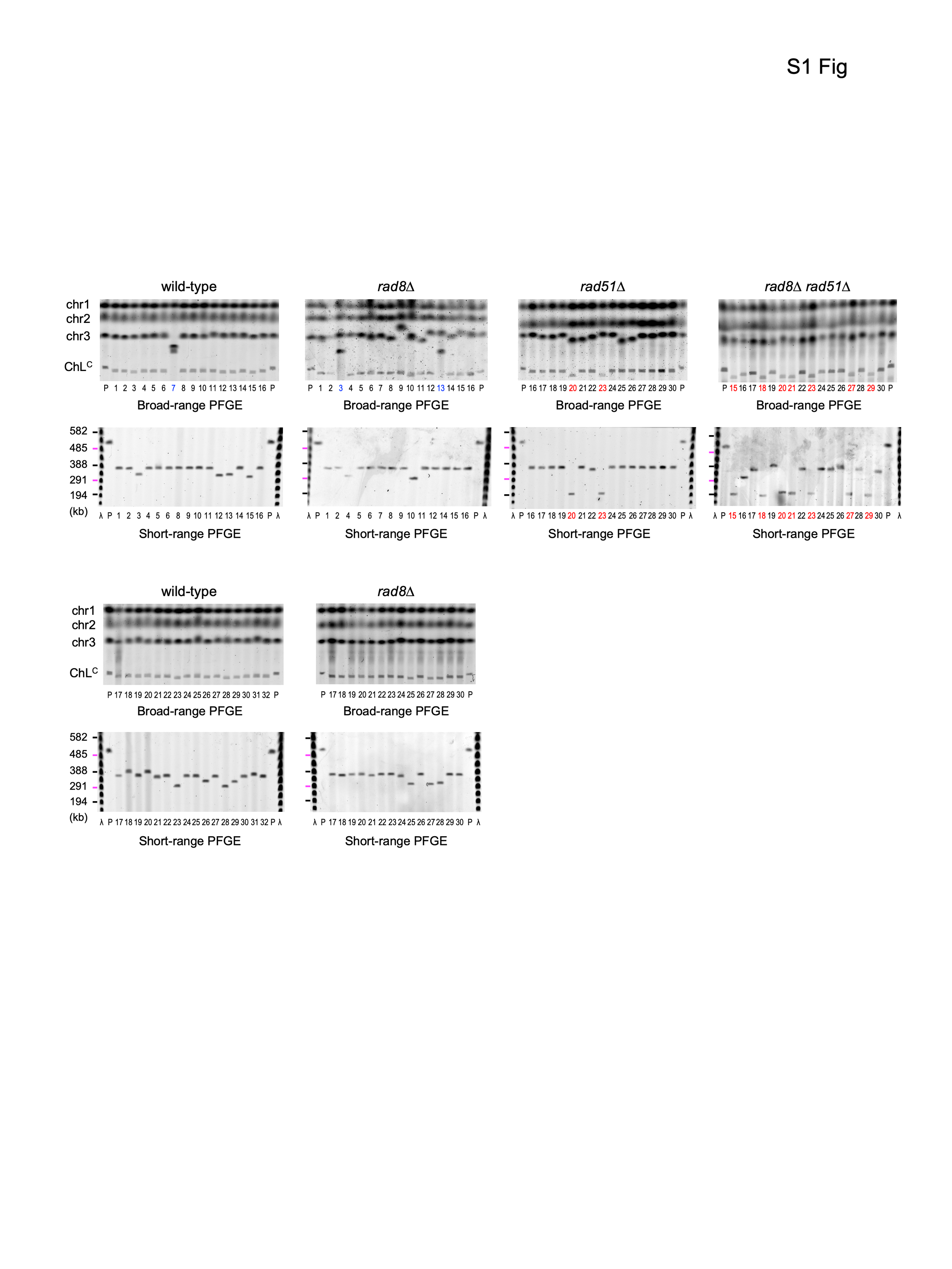

Supplement: S1 Fig — Chromosomal DNAs prepared from the parental and independent GCR clones of the wild-type, rad8Δ, rad51Δ, and rad8Δ rad51Δ strains (TNF5369, 5549, 5411, and 5644, respectively) were separated by broad- and short-range PFGE and stained with EtBr. Sample number of translocations and truncations are highlighted in blue and red, respectively. (TIF) [file pgen.1009671.s003.tif]

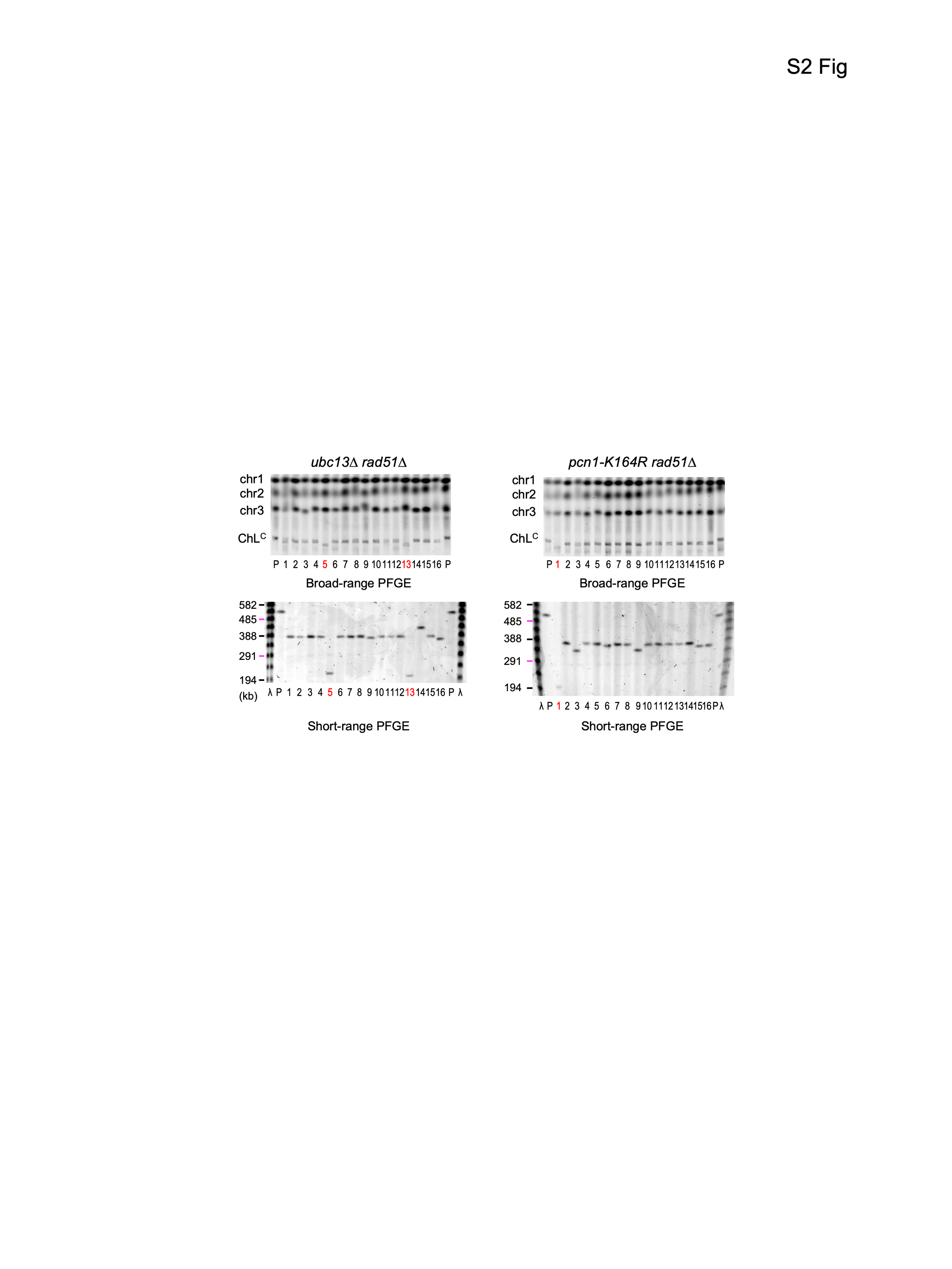

Supplement: S2 Fig — Chromosomal DNAs prepared from the parental and independent GCR clones of the ubc13Δ rad51Δ and pcn1-K164R rad51Δ strains (TNF6115 and 6104, respectively) were separated by broad- and short-range PFGE and stained with EtBr. Short-range PFGE ran at 4.5 V/cm with a pulse time from 4 to 120 s for 48 h at 4°C. (TIF) [file pgen.1009671.s004.tif]

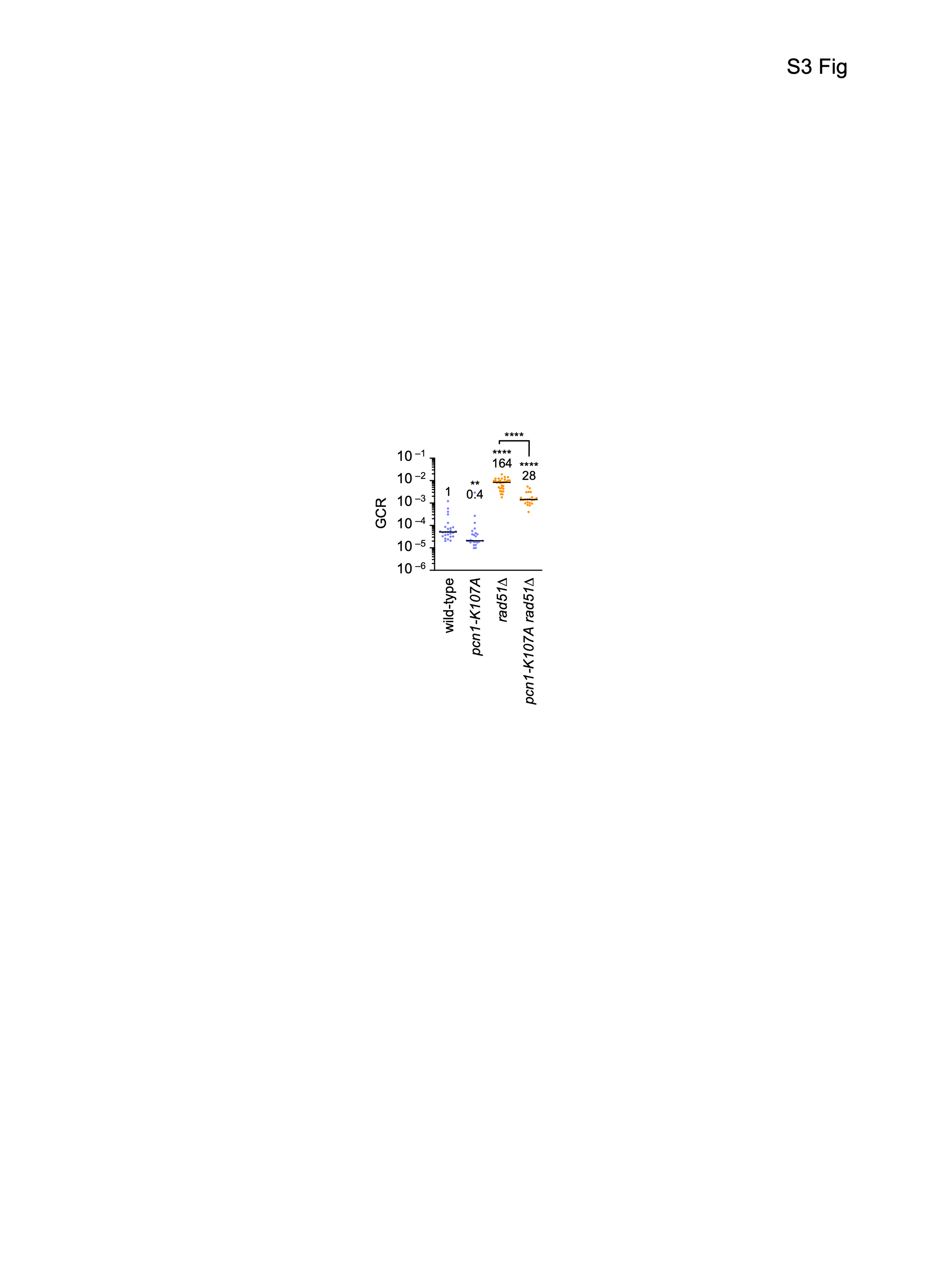

Supplement: S3 Fig — GCR rates of the wild-type, pcn1-K107A, rad51Δ, and pcn1-K107A rad51Δ strains (TNF5369, 6699, 5411, and 6719, respectively). The two-tailed Mann-Whitney test. ** P < 0.01; **** P < 0.0001. Numerical source data underlying the graph are provided in Table M in S1 File. (TIF) [file pgen.1009671.s005.tif]

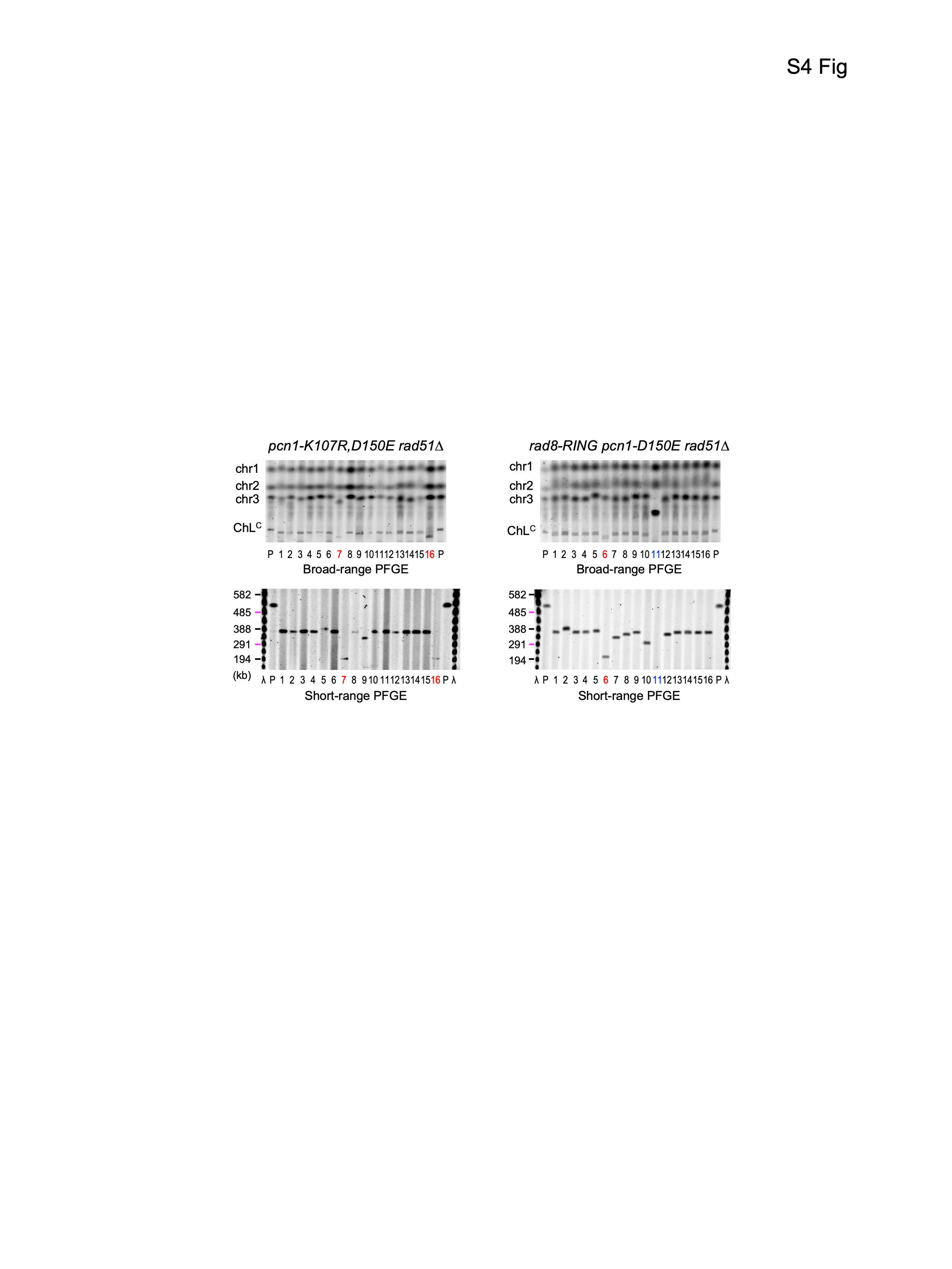

Supplement: S4 Fig — Chromosomal DNAs prepared from the pcn1-K107R,D150E rad51Δ and rad8-RING pcn1-D150E rad51Δ strains (TNF7747 and 7773, respectively) were separated by broad- and short-range PFGE and stained with EtBr. (TIF) [file pgen.1009671.s006.tif]

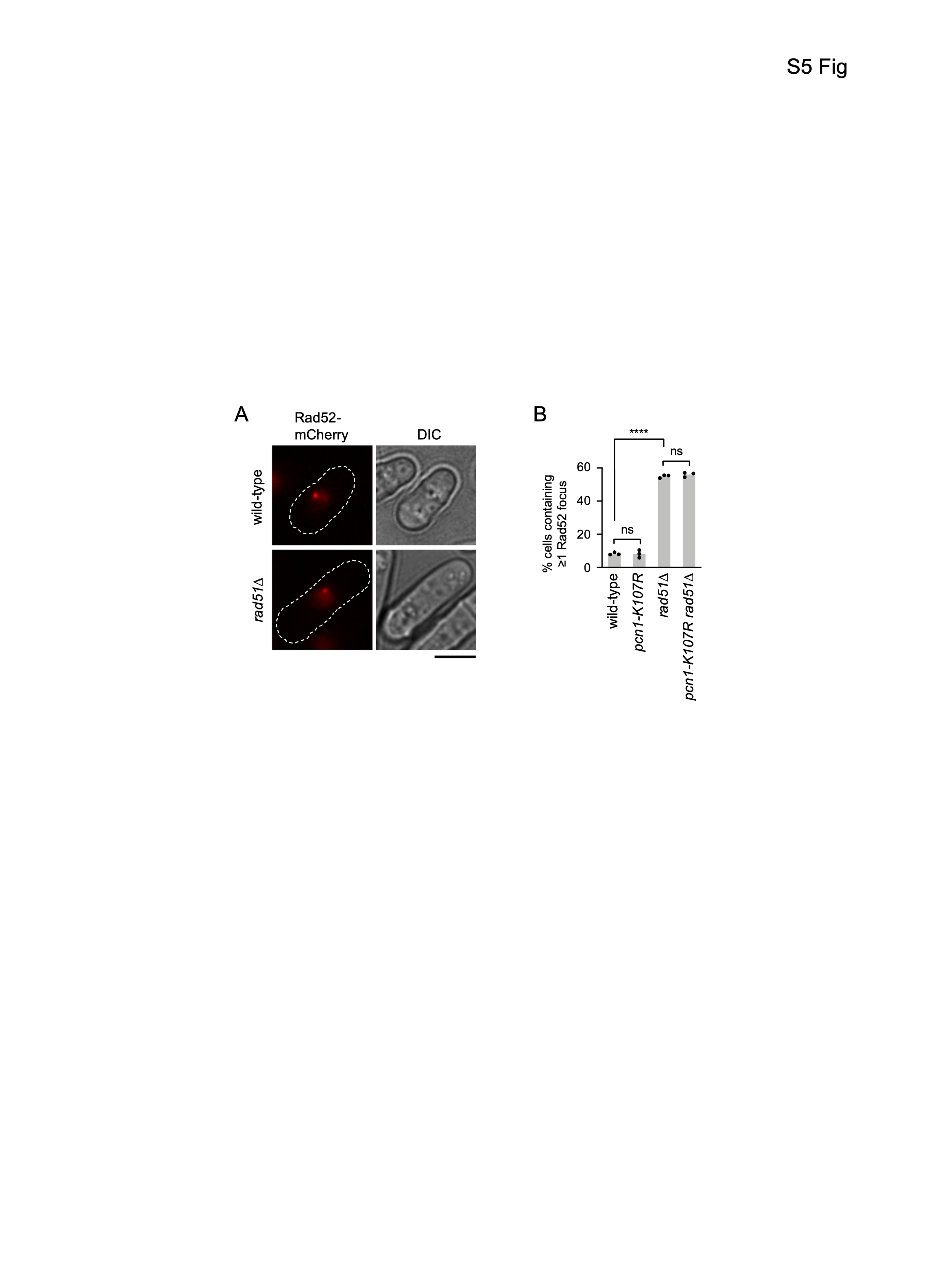

Supplement: S5 Fig — (A) Rad52-mCherry foci were observed by fluorescence microscopy. DIC, differential interference contrast. A scale bar indicates 5 μm. (B) Percentages of cells containing Rad52 foci in the wild-type, pcn1-K107R, rad51Δ, and pcn1-K107R rad51Δ strains (TNF4462, 7387, 7800, and 7802, respectively). Bars represent the mean of three independent experiments shown as dots. > 200 cells were counted in each experiment. The two-tailed student’s t-test. Non-significant (ns) P > 0.05; **** P < 0.0001. Numerical source data underlying the graph shown in (B) are provided in Table N in S1 File. (TIF) [file pgen.1009671.s007.tif]

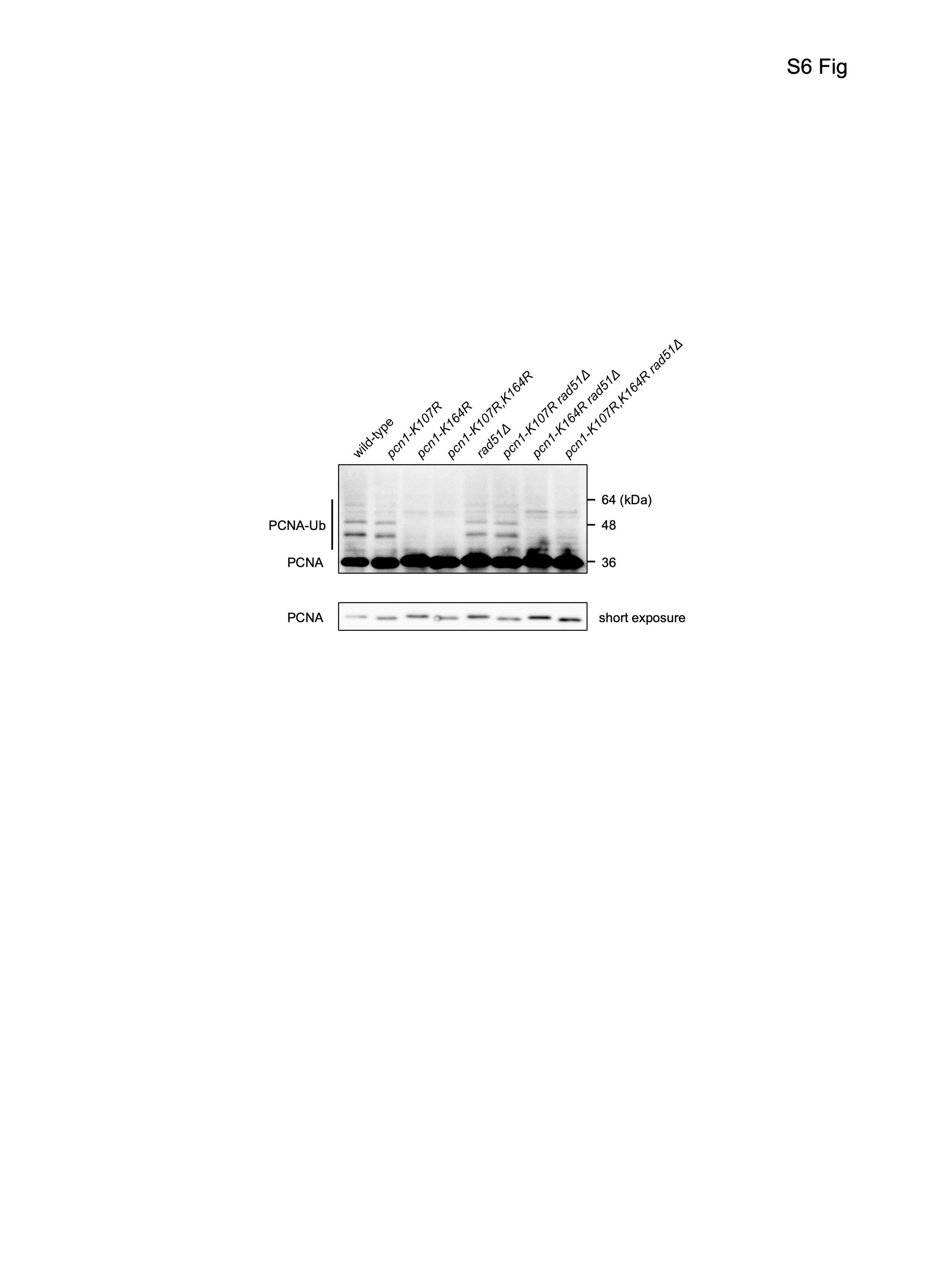

Supplement: S6 Fig — Extracts prepared from the wild-type, pcn1-K107R, pcn1-K164R, pcn1-K107R,K164R, rad51Δ, pcn1-K107R rad51Δ, pcn1-K164R rad51Δ, and pcn1-K107R,K164R rad51Δ strains (TNF35, 6968, 6948, 6996, 2610, 6988, 6986, and 7012, respectively) were resolved by 10% SDS-PAGE. PCNA was detected using anti-PCNA antibodies at 1:2,000 dilution. Sizes of pre-stained protein markers (Nacalai tesque, 02525–35) are indicated on the right of the image. It might be worth noting that the pcn1-K107R mutation increased the mobility of PCNA in SDS-PAGE. (TIF) [file pgen.1009671.s008.tif]

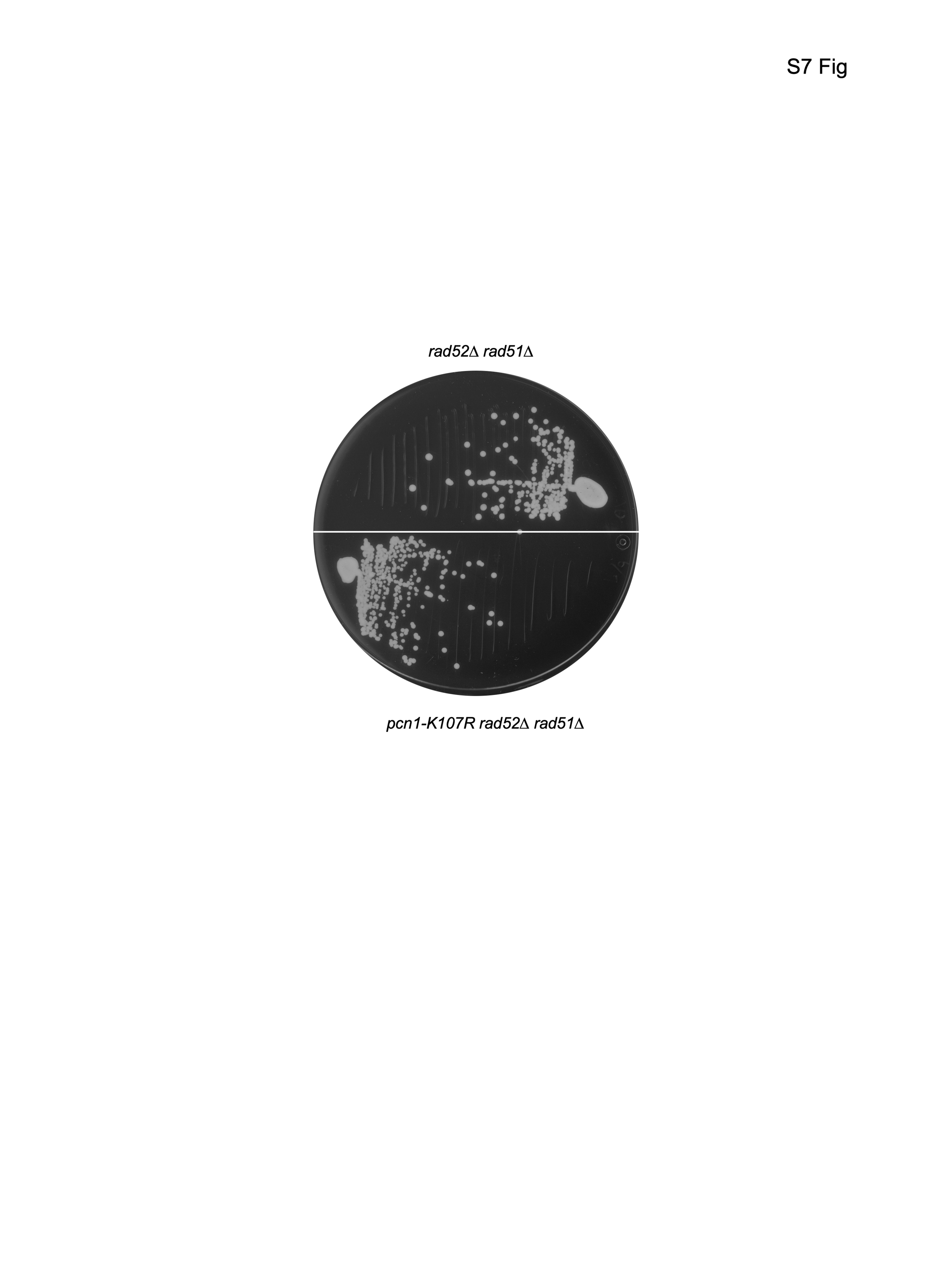

Supplement: S7 Fig — The rad52Δ rad51Δ and pcn1-K107R rad52Δ rad51Δ strains (TNF6692 and 6922, respectively) were incubated on EMM+UA plates. We took a picture of colonies after seven days’ incubation at 30°C. There were no exceptionally large colonies on this plate. (TIF) [file pgen.1009671.s009.tif]
